# A strongly adhesive hemostatic hydrogel for the repair of arterial and heart bleeds

**DOI:** 10.1038/s41467-019-10004-7

**Published:** 2019-05-14

**Authors:** Yi Hong, Feifei Zhou, Yujie Hua, Xianzhu Zhang, Chengyao Ni, Dihao Pan, Yiqing Zhang, Deming Jiang, Long Yang, Qiuning Lin, Yiwei Zou, Dongsheng Yu, David E. Arnot, Xiaohui Zou, Linyong Zhu, Shufang Zhang, Hongwei Ouyang

**Affiliations:** 1 Department of Orthopaedic Surgery, Second Affiliated Hospital and Zhejiang University-University of Edinburgh Institute and School of Basic Medicine, Zhejiang University School of Medicine, Hangzhou, 310003 China; 2 Dr. Li Dak Sum and Yip Yio Chin Center for Stem Cells and Regenerative Medicine, Zhejiang University School of Medicine, Hangzhou, 310003 China; 3 Optogenetics and Synthetic Biology Interdisciplinary Research Center, State Key Laboratory of Bioreactor Engineering, School of Chemistry and Molecular Engineering, East China University of Science and Technology, 130 Mei Long Road, Shanghai, 200237 China; 4 Department of Cardiothoracic Surgery, The First Affiliated Hospital, Zhejiang University School of Medicine, Hangzhou, Zhejiang Province 310003 China; 5 Clinical Research Center, The First Affiliated Hospital, Zhejiang University School of Medicine, Hangzhou, Zhejiang Province 310003 China; 6 China Orthopedic Regenerative Medicine Group (CORMed), Hangzhou, 310003 China; 7 Key Laboratory of Tissue Engineering and Regenerative Medicine of Zhejiang Province, Zhejiang University School of Medicine, Hangzhou, 310003 China; 8 Department of Sports Medicine, Zhejiang University School of Medicine, Hangzhou, 310003 China

**Keywords:** Biomaterials, Trauma, Biomaterials

## Abstract

Uncontrollable bleeding is a major problem in surgical procedures and after major trauma. Existing hemostatic agents poorly control hemorrhaging from traumatic arterial and cardiac wounds because of their weak adhesion to wet and mobile tissues. Here we design a photo-reactive adhesive that mimics the extracellular matrix (ECM) composition. This biomacromolecule-based matrix hydrogel can undergo rapid gelling and fixation to adhere and seal bleeding arteries and cardiac walls after UV light irradiation. These repairs can withstand up to 290 mm Hg blood pressure, significantly higher than blood pressures in most clinical settings (systolic BP 60–160 mm Hg). Most importantly, the hydrogel can stop high-pressure bleeding from pig carotid arteries with 4~ 5 mm-long incision wounds and from pig hearts with 6 mm diameter cardiac penetration holes. Treated pigs survived after hemostatic treatments with this hydrogel, which is well-tolerated and appears to offer significant clinical advantage as a traumatic wound sealant.

## Abstract (web summary)

Uncontrollable bleeding is a major problem in surgery and after trauma. Here the authors design a photo-reactive adhesive that mimics the composition of connective tissue and is able to stop high pressure bleeding within half a minute.

## Introduction

Uncontrollable bleeding following trauma or occurring during surgery is a major cause of global mortality^1^. In particular, repair of aortic rupture and stemming heart bleeding from cardiac penetration wounds are difficult surgical challenges. Currently, surgical suture is the only clinical method for aortic rupture and heart wound sealing; however, this is not feasible outside surgical units and is not attempted in most emergency situations^2^. With recent progress in materials science, many experimental chemical agents have been tested for rapid wound sealing, such as fibrin glue, gelatin, collagen, oxidized cellulose, zeolites, peptides, polymers, and hydrogels^3–9^. However, none of these materials are suitable for aortic and heart trauma hemostasis and sealing because of their slow hemostatic performance, poor wet tissue surface adhesion, and weak or inflexible bonding mechanics.

Rapid hemostasis for cardiac and arterial breeches requires fast adhesion in the presence of continuous blood flow, strong adhesion between wet tissue walls and surfaces, high mechanical strength to maintain blood pressure, and good biocompatibility for tissue regeneration. However, it is extremely difficult to bond wet and dynamic tissue surfaces^10–12^. Cyanoacrylate (CA) is a strong adhesive and has been investigated for vascular anastomosis^13^; however, it is cytotoxic and fails to bond wet surfaces, as it solidifies immediately upon exposure to water^10^. Although a few studies have reported that other artificial polymers can adhere to wet tissue surfaces, the clinical application of those materials has been limited due to either their long gelling time, inflexibility, or toxic degradation products^10,14,15^.

Here we report a biomimetic tissue adhesive product that can polymerize and adhere within seconds, and strongly bond to wet biological tissue surfaces after UV photo-activation. The product design has been inspired by consideration of the extracellular matrix composition of biological connective tissues. These are generally made of collagen (10–15%), glycosaminoglycans (3–6%), and water (70–80%), and have strong and flexible bio-mechanical properties^16^. The matrix hydrogel bio-adhesive used here was composed of 5% methacrylated gelatin (GelMA), 1.25% *N*-(2-aminoethyl)−4-(4-(hydroxymethyl)−2-methoxy-5-nitrosophenoxy) butanamide (NB) linked to the glycosaminoglycan hyaluronic acid (HA-NB)^17^ with 0.1% of the polymerization initiator lithium phenyl-2,4,6-trimethylbenzoylphosphinate (LAP).

The chosen ratio of GelMA:HA-NB in our candidate product is similar to that of collagens to glycosaminoglycans of human connective tissue. In a previous study, this class of HA-NB polymer matrices has shown good tissue fusion and integration, probably due to UV photo-generated aldehyde groups bonding with the amino groups on the tissue surface^17^. However, in this prototype gel, it took more than 20 s for HA-NB to polymerize with other primary amino-bearing macromolecules and unpolymerized material was easily washed away by blood flow before a bonded gel could be formed. The introduction of a GelMA component into the hydrogel maintains its capacity for wet wound sealing and accelerates polymerization by an order of magnitude, thus greatly increasing the clinical utility of this wound sealing hydrogel.

## Results

### Matrix gel synthesis and its physical characterization

The matrix hydrogel bio-adhesive used here was composed of 5% GelMA, 1.25% NB linked to the HA-NB^17^ with 0.1% of the polymerization initiator LAP. We selected these precursor and relative composition ratios in a prior series of optimization and characterization studies on the chemistry and mechanical properties of biomimetic matrix hydrogels (see Supplementary Fig. 1 and Supplementary Table 1).

**Fig. 1:**
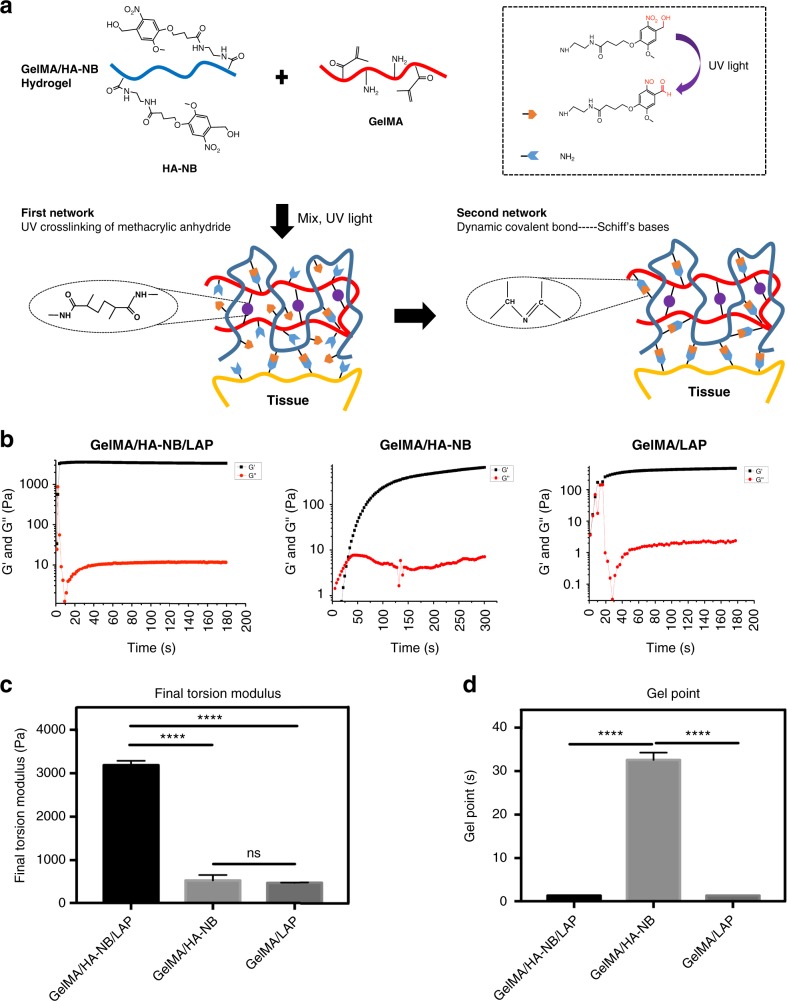
Chemical structure and mechanical properties of the hydrogels. **a** Constituent chemical structures and a schematic diagram illustrating the formation of the photo-triggered imine-crosslinked matrix hydrogel. **b** To monitor the gelling process, a dynamic time-sweep rheological analysis was carried out with an in situ photo-rheometer (HAAKE Mars III, light, Omnicure S2000 365 nm: 30 mW cm^−2^) showing the formation kinetics for GelMA/HA-NB/LAP, GelMA/HA-NB, and GelMA/LAP hydrogels. **c** The final torsion modulus G’ of different hydrogels. **d** The gel point of different hydrogels. All the gelling measurements were conducted using OmniCure S2000 (365 nm, 30 mW/cm^2^). Exposure time: 180 s for GelMA/HA-NB/LAP and GelMA hydrogels, and 300s for GelMA/HA-NB hydrogel (error bars, mean ± SD. ****p *<* 0.0001; NS: no significance, one-way analysis of variance (ANOVA), Tukey’s post hoc test) (*n* *=* 3 per group). Source data are available in the Source Data file

Figure 1a illustrates the chemistry involved in the generation of the hydrogel from HA-NB and GelMA. To overcome the problem of slow polymerization/adhesion, a relatively low-level substituted GelMA (see Supplementary Fig. 2a and Supplementary Table. 1) was chosen, as it will rapidly photo-crosslink internally, while leaving some amino groups available to react with photo-generated aldehyde groups^17^. An ~3.27% substitution level of disaccharides was found to be an optimum compromise between gel strength and important factors such as solubility and biocompatibility (Supplementary Fig. 2b, Supplementary Table. 1, and Supplementary Fig. 1). We found that gels formed in less than a second when exposed to UV (365 nm, 30 mW/cm^2^) (Fig. 1b). To monitor the gelling process, an in situ, dynamic time-sweep rheological experiment was carried out with a photo-rheometer. As shown in Fig. 1b–d, the gel point was reached at 1.384 ± 0.0006 s for GelMA/HA-NB with LAP, similar to the 1.384 ± 0.001 s gel point for GelMA alone with LAP. These are much faster gelling points than the 32.1 ± 1.54 s for GelMA/HA-NB without LAP. The final torsion modulus (twisting shear resistance) after complete gelation is 3183 ± 71 Pa also much larger than the shear resistance of 547 ± 40 Pa for GelMA/HA-NB hydrogels without added LAP and the shear resistance of 475 ± 14 Pa for GelMA with LAP alone (Fig. 1c). These results indicate that the double-network structure of UV-crosslinked GelMA and Schiff bases greatly strengthened the hydrogel, primarily by increasing the degree of internal crosslinking.

The hydrogel formed was porous. The cryo-scanning electron microscopy (cryo-SEM)-observed pore size of GelMA/HA-NB with LAP was nanoscale, smaller, and denser than GelMA/LAP and GelMA/HA-NB. Furthermore, the morphology of GelMA/HA-NB/LAP hydrogel has the characteristics of GelMA/LAP and GelMA/HA-NB hydrogels (Supplementary Fig. 3a). This result is consistent with the formation of two internal networks of UV-crosslinked GelMA and Schiff bases. Maximum swelling ratios (SRs) were reached after 24 h (Supplementary Fig. 3b).

### Adhesion and mechanical strength study of the matrix gel

**Fig. 2:**
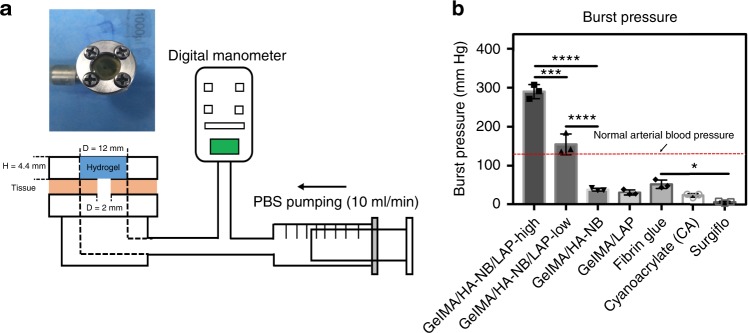
Burst pressure of different hydrogels and surgical hemostatic glues. **a** Schematic illustration of the experimental procedure and pressure chamber for burst adhesion testing using a punctured, then sealed porcine sausage skin membrane. **b** The observed burst pressure of different hydrogels and the commercially available Cyanoacrylate and Fibrin Glue and Surgiflo^**TM**^, a gelatin-based absorbable porcine gelatin paste used for hemostasis (error bars, mean ± SD; *p < 0.05, **p < 0.01, ***p < 0.001, ****p < 0.0001, one-way analysis of variance (ANOVA), Tukey’s post hoc test) (*n* = 3 per group) Source data are available in the Source Data file

The adhesion strength of the hydrogel is one of the most important factors for heart and artery hemostasis, as the hydrogel needs to rapidly adhere to halt bleeding under arterial blood pressure. The adhesive must both form strong bonds with the substrate and the material composition of adhesive plus substrate must dissipate energy by hysteresis^10^. Burst pressure tests are probably the primary efficacy tests relevant to investigating the capacity of these hydrogels to withstand blood pressure, while at the same time rapidly adhering to tissue walls to seal the rupture. In Fig. 2, the capacity of several hydrogels and commercially available surgical glues to adhere to experimental biological surfaces and resist bursting pressures were compared. Hydrogel were formed in situ on the wet surface of porcine sausage skins, covering a 2 mm diameter hole in a chamber linked to a syringe pump and filled with phosphate-buffered saline (PBS) solution. Thirty seconds after UV irradiation to polymerize the hydrogels, the syringe pump began to pump PBS and exert pressure on the hydrogel-sealed hole (Fig. 2a). Figure 2b shows that the measured burst pressure of GelMA/HA-NB with LAP was 155 ± 27 mm Hg, much higher than GelMA /HA-NB without LAP (38 ± 4 mmHg) and GelMA with LAP (31 ± 7 mmHg). The burst pressure of the GelMA/HA-NB with LAP casing seals was significantly higher than the normal systolic blood pressure (120 mm Hg), which would make it a promising sealing agent for hemostasis.

Hemostatic performance fundamentally depends on the weakest property of the materials used and if the adhesion force is higher than the gel’s mechanical strength, the hydrogel could rupture under pulsing blood pressure. In the burst pressure tests, we found the failure positions of GelMA/HA-NB with and without LAP were all directly above the sealed hole (Supplementary Fig. 4a). The hydrogels were punctured but were not separated from the porcine sausage membrane. Using hydrogels without HA-NB led to edge adhesion failures and expulsion of the gel as a plug (Supplementary Fig. 4a). This phenomenon indicated that the tissue adhesion strength of GelMA/HA-NB (with and without LAP) was actually higher than its mechanical strength. We therefore doubled the matrix component concentrations (to 10% GelMA, 2.5% HA-NB, and 0.2% LAP) to enhance hydrogel mechanical strength. The burst pressure resistance increased to 290 ± 18 mm Hg. This value significantly exceeds the performance of all reported adhesives and commercially available surgical sealants^10,15,18^. Two further classes of adhesive strength tests are wound closure tests and peeling adhesion resistance tests. Results of these additional tests are shown in Supplementary Fig. 4b-e. In both wound-sealing and -peeling adhesion assays, at failure, the hydrogel still adheres to the porcine sausage membrane, but the hydrogel itself fractured. Taken together, these material strength tests indicated that it is critical that the adhesive hydrogel has sufficient internal strength and capacity to form strong bonds with the tissue substrate.

More exact data directly determining the chemical composition of the hydrogel and hydrogel–tissue interface can be achieved by X-ray photoelectron spectroscopy (XPS), a technique more commonly employed in material science than in medical biology. Using XPS we detected large-scale formation of C = N bonds on the tissue surface, which supports our hypothesis that Schiff bases are being created at the interface between the tissue and hydrogel (Supplementary Fig. 5). The fact that burst pressure increased as the substitution rates of HA-NB increased also supports this model of extensive Schiff base formation strengthening the hydrogel’s adhesive and mechanical properties (Supplementary Fig. 1 and Supplementary Table 1).

In the process of healing, the wound and matrix gel would be subjected to shear force and mechanical force pressure. Therefore, we used a lap shear test and a compression test to investigate these mechanical properties (Supplementary Fig. 6). Again, these mechanical properties of hydrogels composed of GelMA/HA-NB with LAP were much higher than those composed of GelMA/HA-NB without LAP and GelMA with LAP. These results again demonstrate that the double-network structure of UV-triggered, crosslinked GelMA and Schiff bases significantly strengthens the hydrogel.

### Biocompatibility and biodegradation of matrix gel

Good biocompatibility is an essential property of hemostatic adhesives. The cellular cytotoxicity of these hydrogels was initially evaluated by co-incubation with L929 fibroblastic cells. The hydrogel-exposed cells and the untreated control cell cultures grow (Supplementary Fig. 7c). Fibroblasts were also encapsulated in situ with the hydrogel and cultured for 5 days. The great majority of cells survived this treatment and stained normally (Supplementary Fig. 7a). C3H cells, which easily form spreading monolayers, attach and spread well on the hydrogel surface after seeding, and these cells were also observed to proliferate and migrate into the hydrogel as culture progressed (Supplementary Fig. 7b). Finally, this hydrogel was subcutaneously implanted in rats to assess in vivo biodegradation and local interaction of the implant with the animal tissue and the cellular immune response of the host. Implanted material was excised for assay at days 7, 14, 28, and 56. The size of the implants increased after implantation because of initial osmotic swelling and local inflammation, then decreased with time after implantation as the hydrogel degraded (Supplementary Fig. 8b, c, e). The proportion of implanted mass remaining after 7 days was 82.5 ± 5.5%, after 14 days this had diminished to 59.6 ± 6.7%, after 28 days 25.5 ± 6.7% remained, and after 56 days only 20.0 ± 5.0% remained. These results indicate that the hydrogel progressively biodegrades (Supplementary Fig. 8d).

A hemostatic agent should not compromise wound healing^19^ and surgical sealants, and hemostats must not be toxic or highly inflammatory^20–22^. Pathological staining with hematoxylin and eosin showed that a number of inflammatory cells appeared near the interface of hydrogel and tissue, indicating that, as would be expected, a postoperative inflammatory response occurred. However, this inflammatory response gradually disappeared, and progressive growth and migration of non-inflammatory dividing cells occurred within 2 weeks (Supplementary Fig. 8). Similarly, more severe postoperative inflammation was observed in a Fibrin Glue-treated group, whereas some necrosis was observed from a CA glue-treated group (Supplementary Fig. 8). These results indicate good biocompatibility and diminished cytotoxicity following the hydrogel treatments.

### Sealant and hemostatic performance on rabbits and pigs

Our first investigation of the actual in vitro hemostatic capacity of the hydrogel on mammalian tissues used fresh pig livers to simulate hemorrhages. The best way to present such real-time experimentation is via video recordings. The hydrogel is shown to be capable of immediately arresting puncture lesion bleeding following fixation by UV irradiation (Supplementary Movie 1). In a comparative trial, commercially available fibrin glue was washed away by the blood flow and the bleeding could not be stopped (Supplementary Movie 2). These experimental operations vividly demonstrate the importance of fast wound sealing and strong adhesion to rapidly halt blood loss from wet, slippery, and mobile mammalian organs.

**Fig. 3:**
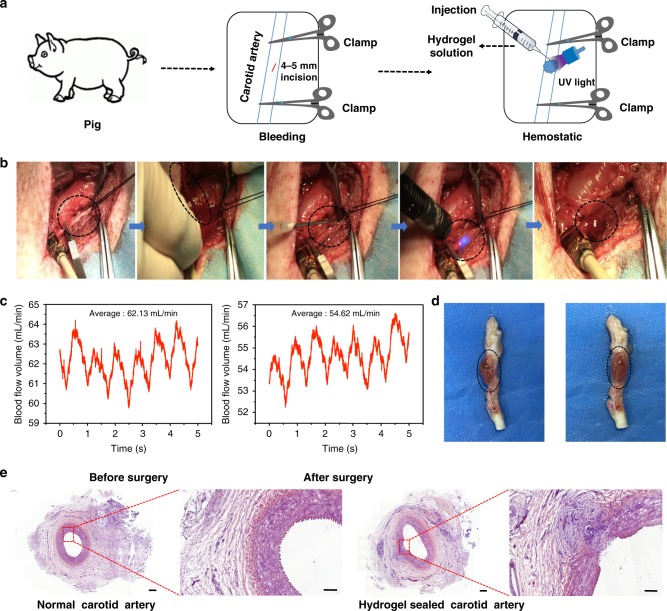
Hemostatic properties of the matrix gel in a pig carotid artery damage model. **a** Schematic diagram of the surgical procedure. **b** Gross view of the rapid hemostasis and sealing in a pig carotid artery model. **c** The blood flow volumes through the pig’s carotid artery before and after surgery. **d** Macroscopic view of a healing hydrogel-covered carotid artery, 2 weeks after operating. **e** Postoperative tissue sections stained with hematoxylin–eosin, showing normal carotid artery (left) and the hydrogel-repaired vessels (right). Scale bar: 500 μm (left plates); 200 μm (right plates, enlarged) (*n* = 3) Source data are available in the Source Data file

In vivo hemostatic capacity tests were performed in both rabbit and pig experimental surgery. In rabbit surgical testing, the matrix gel could stop bleeding of a liver cut and a femoral artery section in a few seconds. Blood losses following hydrogel sealing were significantly less than in the untreated controls and Fibrin Glue wound treatment groups (Supplementary Fig. 9). During pig experimental surgery, 4~ 5 mm punctures were created in the carotid artery, resulting in arterial blood expulsion from the puncture. Hemostatic forceps were then used to clamp the blood vessels and hydrogel was applied, followed by UV irradiation for 3~ 5 s. After 30 s (a conservative precaution to allow complete polymerization), the hemostatic forceps were removed and no further blood leakage was observed (Fig. 3 and Supplementary Movie 3). Because of the vessel and tissue edema caused by injury, the volumetric flow rate decreased after surgery; however, the measured volumetric flow rate indicates the systemic integrity of blood flow has been maintained (Fig. 3c).

We also used Fibrin Glue and Surgiflo^**TM**^ in experimental surgical comparison (Supplementary Movie 4). In this video, it can be seen that when the hemostatic forceps were relaxed, blood flow out immediately when Fibrin Glue or the Surgiflo^**TM**^ had been applied. When the matrix hydrogel was applied, no such bleeding was observed after the hemostatic forceps were removed. All hydrogel-treated pigs survived surgery and were reared for 2 weeks longer (*n* = 3). The hydrogel still adhered to the defect sites after 2 weeks (Fig. 3d) and no thrombus was observed in the vessel, as confirmed by tissue section staining (Fig. 3e). The regeneration of the wound was incomplete, as 2 weeks is a short healing period. However, the wound was connected by neo-vascularized tissues, indicating that healing had commenced. This experimental surgery vividly demonstrates that the matrix gel has the required rapid in vivo hemostatic capacity and can protect major wounds during the postoperative healing process.

The heart is the central organ of vertebrate circulatory physiology and when cardiac penetration injuries occur, massive hemorrhage results in lives being lost within a very short time. We have been able to demonstrate that the hydrogel can be used to close and seal cardiac penetration injuries. A high concentration matrix gel (10% GelMA, 2.5% HA-NB, 0.2% LAP) was used in these experimental surgical procedures to withstand the high blood pressure exerted by heart muscle.

**Fig. 4:**
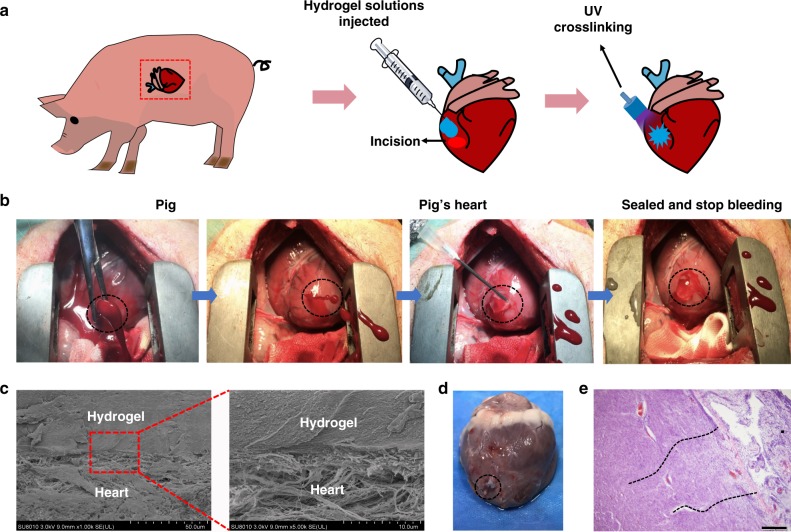
Hemostatic properties of the matrix gel in a pig cardiac puncture injury model. **a** Schematic diagram of the surgical procedure. **b** Gross view of the rapid hemostasis and sealing following cardiac puncture injury: the ventriculus sinister of the pig hearts was pierced by a 6 mm (inner diameter) needle, causing immediate high-pressure blood expulsion, subsequently continuing blood expulsion following needle removal. Then, the matrix gel was injected to cover the blood hole and rapidly irradiated with UV. After UV-induced rapid polymerization, the bleeding stopped completely within 10 s (four experimental operations were carried out). **c** Scanning electron micrographs of the interface between the pig heart puncture wound and the hydrogel. These sections derive from immediate postoperative autopsy on the heart of one pig, which was immediately killed after successful hemostatic treatment. Scale bar: 50 μm (left plates); 10 μm (right plates, enlarged). **d** Images of a heart autopsy following killing after two 2 weeks of postoperative recovery, the hydrogel still adhering to the wound, without any gap between gel and tissues, indicating continuous strong bonding at the healing interfaces. **e** Tissue staining images of the interface between pig heart cardiac tissue and the matrix gel, after 2 weeks of postoperative recovery. Scale bar: 200 μm (*n* = 4) Source data are available in the Source Data file

The surgical operation procedure was illustrated in Fig. 4a, with the ventriculus sinister of the pig heart being pierced by a 6 mm inner diameter needle, resulting in immediate high-pressure blood expulsion (Fig. 4b). Hydrogel was immediately injected to cover the blood hole and UV irradiated to fix and seal the heart muscle puncture wound (Fig. 4b). Although the puncture hole is covered by the gel, blood initially continued to hemorrhage. However, after the UV polymerization, the bleeding decreased rapidly and soon stopped completely (Fig. 4b). The hemostatic process took <30 s (Supplementary Movie 5). High-pressure ventricular bleeding occurred after the large diameter needle was removed during some of these surgical procedures (Supplementary Movie 6). Even in such serious cardiac wounds, the bleeding was stopped by application of the matrix gel within 30 s (Supplementary Movie 6). In similar situations, 4 mL Fibrin Glue and 8 mL Surgiflo^TM^ were injected to the wound, but failed to stop such bleeding, which was only stemmed by application of the matrix gel (Supplementary Movie 7).

**Fig. 5:**
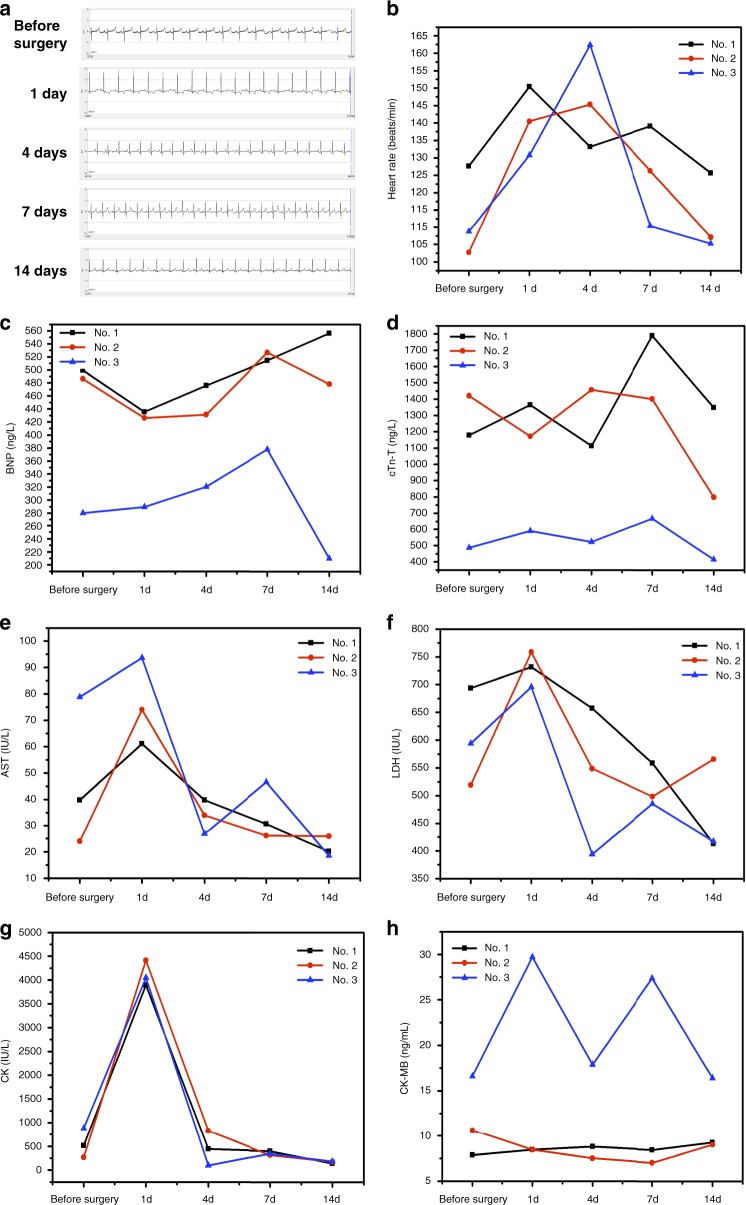
Pre-and postoperative physiological indices in treated pigs. **a** Electrocardiogram (ECG) of a pig (No. 2) before and after surgery (1, 4, 7, and 14 days after treatment). **b** The heart rate of pigs before and after surgery. **c** The level of brain natriuretic peptide (BNP), before and after surgery (1, 4, 7, and 14 days after treatment). **d** Cardiac Troponin T (cTn-T), before and after surgery (1, 4, 7, and 14 days after treatment). **e** Aspartate aminotransferase (AST), **f** lactate dehydrogenase (LDH), **g** creatine kinase (CK), and **h** creatine kinase-MB (CK-MB) in the blood of pigs, sampled after their cardiac surgery (*n* = 3) Source data are available in the Source Data file

Postoperative analysis of cardiac tissues after autopsy was carried out on one pig. SEM analysis was used to investigate the ultrastructural appearance of the zone of matrix gel adhesion to heart tissue, immediately after experimental surgery, following killing of one pig (Fig. 4c). The appearance of closed, effective sealing indicates that the procedure has been successful. Three other pigs were allowed to recover after surgery and were kept for a further 2 weeks. The electrocardiogram of pig 2 showing no abnormalities during the 2-week nursing period (Fig. 5a). The heart rate of the pig was elevated slightly for the first week after surgery, possibly caused by anesthesia and operative shock, but had returned to normal by the second week (Fig. 5b).

The level of blood aspartate aminotransferase (AST), lactate dehydrogenase (LDH), and creatine kinase (CK) rose after surgery, indicative of the trauma caused by operations (Fig. 5e–g). These indices returned to normal levels after 4 days of recovery. Levels of brain natriuretic peptide (BNP), cardiac troponin T (cTn-T), and CK-MB in the blood did not rise significantly, indicating that no myocardial infarction or heart failure happened during the 2-week recuperation period (Fig. 5c–h). This cardiac marker analysis indicates the resumption of normal heart function in these pigs after surgery. The hydrogel also effectively sealed the heart wound after 2 weeks (Fig. 4d), and almost no necrosis and very little inflammation were observed at the wound interface in pathology section staining (Fig. 4e), confirming the excellent biocompatibility of the matrix hydrogel.

## Discussion

It is the first time that high-pressure bleeding of beating heart with 6 mm diameter cardiac penetration holes were rapidly stopped and the wounds were stably sealed by only using matrix gel within 20 s without suture. Although some studies have reported other polymer-based materials could adhere on wet tissue surface^10,14^, suture is still needed for pre-closure of the wound before closure with the material or the materials that are not safe for clinical use, as the degradation products are toxic. We gave these hydrogels excellent functions, e.g., fast wound sealing, strong wet and dynamic tissue surface adhesion, suitable mechanical properties with good biocompatibility, and, importantly, ease of use, through modification and combination of the natural bio-macromolecules. All this is not simple to achieve.

Our data demonstrate that this synthetic gel with its controllable and rapid matrix polymerization properties can rapidly stop bleeding from cardiac penetration injuries. The effective wet-adhesion properties of matrix gel derive from photo-generated aldehyde groups bonding with the amino groups on the tissue surface and its mechanical strength derives from its two internal covalently crosslinked networks. Upon UV irradiation, the GelMA rapidly generates the first crosslinked network elements of the hydrogel. Meanwhile, some of the photo-generated aldehyde groups on HA-NB at the tissue–hydrogel interface react with amino groups of the tissue proteins and proteoglycans, forming Schiff bases. Meanwhile, the photo-generated aldehyde groups on the hydrogel HA-NB react with the amino groups of GelMA to form a second crosslinking network. As the reaction progresses, the remaining aldehyde groups react with amino groups of tissue and GelMA to bond tissue and increase internal crosslinking. The creation of two different chemically crosslinked matrices leads to a significant increase in the tissue adhesion and internal strength of the hydrogel^23^. The good wet tissue adhesion and mechanical properties of the hydrogel would ensure its stability in wound closure.

Maintaining porous structures in hydrogels intended to be used as hemostatic agents is important in stopping bleeding, as dense, porous material can absorb wound exudates and largely block escape of red blood cells and platelets^24^, while maintaining a suitably moist environment for effective wound healing^25,26^. Although hydrogels will absorb water from the serum to concentrate red blood cells and platelets^24^, large SRs could compromise the mechanical strength of the hydrogel, causing the hydrogel to break before wound healing can take place^27^. These GelMA/HA-NB/LAP hydrogels appear to have suitable SRs, which can meet the requirement for effective absorption of water from serum, while maintaining mechanical sealing and protection of the wound.

Here we need to declare that blank control in pig carotid artery model cannot be used, because bleeding could not be stopped and the pig would die in few minutes because of severe blood loss. In this kind of experimental surgery, we think that comparative quantification of the blood losses is not really possible, as the animals would simply not survive control or inadequate treatments. No more bleeding was observed in matrix gel-treated groups in our surgical experiments but this was not the case in the other treatment groups (Supplementary Movie 4).

For the penetrated cardiac injuries hemostatic experiments, we have tried to use an aspirator to collect blood during the operation. However, this is a not very accurate process because it is difficult to collect all the blood. We also weighed the pig before and after surgery as to supplement this data, but this is also inaccurate because unanticipated excretions happened in some pigs during the operation. What is relevant is that most of the blood loss occurred before the matrix gel was used and almost no blood was lost after the application of the matrix gel (see Supplementary Movies 5–7). In Supplementary Movie 7, neither Fibrin Glue nor Surgiflo^TM^ could staunch bleeding. Taking these facts into consideration, we think true quantitative comparisons of the blood losses are not realistically possible in these penetrated cardiac injuries hemostasis experiments. Bleeding was staunched in matrix gel group yet the bleeding was not controlled and the animal would die in the Fibrin Glue, Surgiflo^TM^-treated, and untreated groups.

The hemostatic and wound-sealing performance of matrix gel is significantly better than commercial Fibrin Glue and Surgiflo^TM^. Together with its biomimetic composition and biocompatibility, these properties make the matrix hydrogel a radically improved hemostatic sealant. Meanwhile, safety is the top priority in the clinical application of biomaterials. The matrix gel exhibit excellent biocompatibility in both in vitro and postoperative recovery. However, these studies are only the preliminary steps in safety testing. In further studies, we are carrying out more in-depth research to assess whether our hydrogel has any potential toxicity before it is applied to humans.

Numerous attempts have been made to develop alternatives to suturing because of the difficulty and limitations of suture techniques when dealing with diseased, damaged, or small blood vessels^15,28–30^. The precursor of this matrix gel has relatively low viscosity and can be readily injected. Meanwhile, light-induced polymerization is an accurate and convenient method that have been widely used in dental medicine. With a UV flashlight or an optical fiber, this material can potentially be used for completely sutureless hemostasis and sealing, which make it a promising bio-glue for use in surgery and emergency hemostasis.

## Methods

### Synthesis of NB and HA-NB

Methyl 4-(4-(hydroxymethyl)−2-methoxy-5-nitrophenoxy)butanoate (mNB) was synthesized as follows^17^: 4-hydroxy-3-methoxybenzaldehyde (vaniline) (8.90 g, 58.5 mmol, 1.06 eq.), methyl 4-bromobutanoate (9.89 g, 55.0 mmol, 1.0 eq.), and potassium carbonate (10.2 g, 73.8 mmol, 1.34 eq.) were dissolved in N, N-Dimethylformamide (DMF) (40 mL). The mixture was stirred at ambient temperature for 16 h, after which time the resulting solution was poured into chilled water (200 mL) and allowed to precipitate for 15 min at 0 °C. The solid was filtered off, washed with water, redissolved in dichloromethane, and dried over magnesium sulfate. The solvent was removed under reduced pressure to yield a white solid (methyl 4-(4-formyl-2-methoxyphenoxy)butanoate, 12.2 g, 48.4 mmol, 88 %). Methyl 4-(4-formyl-2-methoxyphenoxy)butanoate (9.4 g, 37.3 mmol, 1 eq.) was added slowly to a pre-cooled (−2 °C) solution of nitric acid (70%, 140 mL) and stirred at −2 °C for 3 h. It is important to note that—depending on the temperature of the nitration reaction—ipso substitution of the formyl moiety occurs. The resulting solution was poured into chilled water (500 mL) and allowed to precipitate for 15 min at 0 °C (note: as saponification of the product can occur under these conditions, the precipitation time should be kept as short as possible). The product was filtered, washed with water, and dissolved in dichloromethane. The organic layer was dried over magnesium sulfate. The solvent was removed under reduced pressure to yield a slightly yellow powder (methyl 4-(4-formyl-2-methoxy-5-nitrophenoxy)butanoate, 7.7 g, 25.9 mmol, 69%). Sodium borohydride (1.50 g, 39.7 mmol, 1.5 eq.) was slowly added at 0 °C to a solution of methyl -(4-formyl-2-methoxy-5-nitrophenoxy)butanoate (7.7 g, 25.9 mmol, 1.0 eq.) in EtOH/THF 1:1 v/v (100 mL). After 3 h, all solvents were removed in vacuo and the residue was suspended in water (50 mL) and dichloromethane (50 mL). The aqueous layer was extracted two times with dichloromethane (2 × 50 mL) and the combined organic layers were dried over magnesium sulfate. The solvent was removed under reduced pressure. In order to increase the overall yield and to remove partially saponified products, methanol (100 mL) and tosylic acid (50 mg) were added to the residue. The solution was stirred at room temperature overnight. The solvent was removed in vacuo and the residue was suspended in water (50 mL) and dichloromethane (50 mL). The aqueous layer was extracted two times with dichloromethane (2 × 50 mL) and the combined organic layers were dried over magnesium sulfate. The solvent was removed under reduced pressure to yield a yellow solid as a raw product, which was purified by column chromatography on silica gel using hexane/ethyl acetate = 1:1 (*R*f = 0.6) and finally 5.22 g (4.81 mmol, 75%) of a slightly yellow powder mNB were obtained.

Then mNB (0.5 g, 1.8 mmol) and ethylenediamine (1.1 mL, 2 mmol, Sigma-Aldrich) were dissolved in methanol. The mixture was refluxed overnight until the starting individual components were undetectable by thin layer chromatography. After the reaction was complete, the solvent was evaporated under vacuum. The crude precipitate was dissolved in methanol and re-precipitated three times using ethyl acetate. The filter cake was then dried for 12 h at 30 °C under vacuum until NB appeared as light yellow powder (0.4 g, 1.2 mmol, 66.7%). HA-NB was synthesized according to a previous report^17^. Briefly, HA (408 mg, 1 mmol of disaccharide unit, Dongyuan Biotech, Zhenjiang) was dissolved in 50 mL deionized water at room temperature and NB (224 mg, 0.69 mmol) was added followed by HOBt (153 mg, 1 mmol, Sigma-Aldrich). The pH of the mixed solution was adjusted to pH 4.5, after which 1-(3-Dimethylaminopropyl)–3-ethylcarbodimide hydrochloride (200 mg, 1.04, Sigma-Aldrich) was added to the mixture and stirred for 48 h at room temperature. The solution was loaded into dialysis tubing (Molecular Weight (MW) cutoff 3500, Spectrum®) and dialyzed against diluted HCl (pH 3.5) containing 0.1 M NaCl for 2 days, then dialyzed against deionized water for a further 2 days. The solution was lyophilized and HA-NB was obtained in powder form. The substitution degree of nitrobenzyl groups (3% of HA disaccharide units) was verified by ^1^H-nuclear magnetic resonance (NMR).

### Synthesis of GelMA

Type A gelatin (Sigma-Aldrich) was dissolved in PBS at 50 °C to make a 10% w/v homogeneous solution. Then, a 0.1 mL methacrylic anhydride (MA) (Sigma-Aldrich) per gram of gelatin was added to the gelatin solution at a rate of 0.5 mL/min, with continuous stirring. The mixture was allowed to react at 50 °C for 3 h. The GelMA solution was dialyzed against deionized water using 8–14 kDa cutoff dialysis tubing (VWR Scientific USA) for 6 days at 50 °C to remove unreacted MA and any byproducts. The GelMA solution was frozen overnight at −80 °C, then lyophilized and stored at −20 °C until further use^31,32^. The substitution degree of MA was verified by ^1^H-NMR.

### Synthesis of the photo-initiator

Dimethyl phenylphosphonite (Ourchem) was reacted with 2,4,6-trimethylbenzoyl chloride (Sigma-Aldrich) via a Michaelis–Arbuzov reaction. At room temperature and under argon gas, 3.2 g (0.018 mol) of 2,4,6-trimethylbenzoyl chloride was added dropwise to an equimolar amount of continuously stirred dimethyl phenylphosphonite (3.0 g). The reaction mixture was stirred for 18 h whereupon a fourfold excess of lithium bromide (Aladdin, 6.1 g) in 100 mL of 2-butanone (Sinopharm Chemical Reagent) was added to the reaction mixture from the previous step, which was then heated to 50 °C. Ten minutes later, a solid precipitate had formed. The mixture was cooled to ambient temperature, allowed to rest for 4 h, and then filtered. The filtrate was washed and filtered three times with 2-butanone to remove unreacted lithium bromide and excess solvent was removed by vacuum^33^.

### Precursor preparation of the hydrogels

For precursors of GelMA/HA-NB/LAP hydrogel, the freeze-dried GelMA foams and HA-NB foams were dissolved in PBS solution at 40 °C, then the photo-initiator LAP was added. The final precursor is composed of 5% GelMA, 1.25% HA-NB, and 0.1% LAP for low-concentration matrices, and 10% GelMA, 2.5% HA-NB, and 0.2% LAP for high-concentration matrices. For precursors of GelMA/HA-NB hydrogels, the freeze-dried GelMA foams and HA-NB foams were dissolved in PBS solution at 40 °C to a final concentration of 5% GelMA and 1.25% HA-NB. For precursors of GelMA/LAP hydrogels, the freeze-dried GelMA foams were dissolved in PBS solution at 40 °C and then added to the photo-initiator LAP to a final concentration of 5% GelMA and 0.1% LAP.

### Adhesion mechanism study

XPS (Kratos AXIS Ultra DLD) was used to characterize the surface composition of the sausage skin membranes; skins were treated with non-photo illuminated HA-NB and also with photo-illuminated HA-NB, using an Al Kα source (1486.6 eV). A detailed scan for Nitrogen was carried out with a step of 0.1 eV. The Carbon 1*s* peak (284.6 eV) was used for calibration.

### SEM analysis

Cryo-SEM imaging was performed on a FEI Helios NanoLab 600i Analytical Field Emission Scanning Electron Microscope fitted with low-temperature sample carrier Quaroum PP3000T, to examine the gross morphology hydrogels. The samples were loaded on the cryo-specimen holder and cryo-fixed in slush nitrogen (−210 °C), then quickly transferred to the cryo-stage in the frozen state. The sample was transferred into the cryo-stage (Quorum Technologies, PP300T, East Sussex, UK), a chamber attached to the microscope (FEI, Helios Nanolab 600i, Hillsboro, USA). Once the sample was inside the chamber, a fracture of the sample was made to get a fresh clean surface to be examined. The temperature of the sample was raised by heating the holder to −90 °C for 30 min, in order to increase the contrast and sublimate free-water in the solid-state lakes, followed by a temperature decrease to −180 °C, to stabilize the sample. The surface of the frozen preparation was then coated with platinum (10 mA, 30 s) to prevent charging of the sample and to obtain a good relation between signal and noise. The coated sample was thereafter transferred into the microscope chamber where it was analyzed at a temperature range of −180 °C. To evaluate the integration of the tissue and hydrogels, the samples were first fixed with 2.5% glutaraldehyde in phosphate buffer (0.1 M, pH 7.0) for >4 h, then washed three times in phosphate buffer for 15 min each. The samples were dehydrated by a graded series of ethanol extractions (30%, 50%, 70%, 80%, 90%, 95%, and 100%) for 15–20 min at each step and the dehydrated samples were coated with gold-palladium in a Hitachi E-1010 ion sputter for 4–5 min before viewing. Then the samples were observed under a SEM (Hitachi TM-1010, Japan).

### SR of the hydrogels

The GelMA/HA-NB/LAP (*n* = 3), GelMA/HA-NB (*n* = 3), and GelMA/LAP hydrogels (*n* = 3) were incubated in PBS at 37 °C for 24 h, then lightly blotted dry and weighed (Ws). Hydrogels were then freeze-dried and weighed to determine the dry weight (Wd). The SR of the swollen gel was calculated according to the following equation^34^.

$${\mathrm{SR}} = \frac{{({\mathrm{Ws}} - {\mathrm{Wd}})}}{{{\mathrm{Wd}}}}$$

### Rheological studies

Rheological properties of hydrogels were analyzed following a reported method^17^. In brief, dynamic rheology experiments were performed using a HAAKE MARS III photo-rheometer with parallel-plate (P20 TiL, 20-mm diameter) geometry and OmniCure Series 2000 (365 nm: 30 mW•cm^−2^) at 37 °C. Time-sweep oscillatory tests of GelMA/HA-NB/LAP (*n* = 3), GelMA/HA-NB (*n* = 3), and GelMA/LAP hydrogels (*n* = 3) were performed at a 10% strain, 1 Hz frequency, and a 0.5 mm gap (CD mode). Strain sweeps were performed on the pregel solution to verify the linear response. The gel point was determined at the time when the torsion modulus (G’) surpassed the loss modulus (G”).

### Burst pressure test

Burst pressure testing was performed using a published method^35^. Briefly, a piece of 4 × 4 cm porcine sausage skin membrane was cut and cleaned to remove any excess fat. The membrane was fixed to the measurement device linked to a syringe pump filled with PBS solution. A 2 mm incision was made on the sausage skin membrane surface and the membrane surface was kept wet. Then, 500 μL precursor solutions were injected onto the incision, after which the hydrogels formed in situ on the puncture site after UV illumination. The thickness of the hydrogels was ~4.4 mm and burst pressure was measured after gel formation. Peak pressure before pressure loss was considered the burst pressure. All measurements were repeated three times. Fibrin Glue (Shanghai RAAS Blood Products, Co., Ltd, Shanghai, China), CA (Beijing Compont Medical Devices, Co., Ltd, Beijing, China), and Surgiflo^TM^ (Ethicon, Inc., Somerville, NJ) were tested using the same parameters and conditions.

### Wound-closure tests

The adhesion strengths of the GelMA/HA-NB/LAP hydrogels and of Fibrin Glue, CA, and Surgiflo^TM^ were tested using the modified ASTM F2458–05 standard, a standard test for the determination of tissue/sealant material adhesive strength^15^. Porcine skin was prepared from fresh porcine skin pieces obtained from a local slaughterhouse. The skin sample dimensions were 30 mm × 10 mm. Tissues were pre-wet by immersion in PBS before testing, then fixed onto two glass slides (25 mm × 50 mm) using the Parafilm^TM^. The tissue was cut in the middle with a straight edge razor to simulate wounding, hydrogel solutions (1 mL) were injected onto the desired adhesion zone (20 × 20 mm), and crosslinked by UV. The two glass slides were placed into an Instron mechanical tester (Instron-5543 with a 1 kN sensor) for adhesion strength test by tensile loading with a strain rate of 1 mm/min. Maximum adhesive strength of each sample was obtained at the point of tearing. All measurements were repeated three times.

### Peeling adhesion test

The peeling adhesion test was performed according to reported methods^10^. The sausage skin membrane was bonded to a rigid polyethylene terephthalate (PET) film with CA glue. One end of the PET film was kept open, in order to limit deformation at the crack tip. Then, hydrogel solutions (1 mL) were injected on the membrane surface (40 × 15 mm) and a hydrogel polymerized by UV irradiation. Finally, another PET film was bonded to the hydrogel (40 × 15 mm) with CA, forming a bilayer with an edge crack. This experimental set-up was used, because the sausage membranes are not transparent to UV and the hydrogel could not form if these membranes were used on both sides. An Instron machine (Instron-5543 with a 1 kN sensor) was used to apply unidirectional tension, while recording the force and the extension. The loading rate was kept constant at 1 mm/min. All measurements were repeated three times.

### In vitro lap shear test

The lap shear test was performed according to a previous study^15^. The shear resistance of the GelMA/HA-NB/LAP hydrogels (*n* = 4), GelMA/HA-NB hydrogels (*n* = 4), and GelMA/LAP hydrogels (*n* = 4) was tested according to the modified ASTM F2255-05 standard for lap shear strength property of tissue adhesives. The sausage skin membrane was bonded to glass slides with CA glue. Then, hydrogel solutions (200 μL) were injected on the membrane surface (10 mm × 15 mm) and a hydrogel polymerized by UV irradiation. Finally, another glass slide was bonded to the hydrogel (10 mm × 15 mm) with CA. The two glass slides were placed into an Instron mechanical tester for shear testing by tensile loading with a strain rate of 1 mm/min. The sealant shear strength was determined at the point of detachment.

### Compression test

The compressive stress–strain measurements were performed using a tensile-compressive tester (Instron-5543 with a 1 kN sensor). In compression-crack test, compressive samples were prepared in the molds for compression tests (10 mm in diameter and 5 mm in depth). Prior to the test, hydrogels were incubated in PBS at 37 °C for 4 h. The compressive strain rate was 5 mm/min and strain level was up to 75% of the original height. The compressive moduli were the approximate linear fitting values of the stress–strain curves in the strain range of 15–25%. All measurements were repeated three times.

### Cell encapsulation and proliferation assay

L929 fibroblast cells (L929, Cell bank of the Chinese Academy of Science), at a density of 4 × 10^6^ cells/mL were suspended in the sterile polymer precursor solution GelMA/HA-NB/LAP to evaluate the cytotoxicity of hydrogels. The cell-containing precursor solution (25 μL) was irradiated (30 mW/cm^2^, 3 min) to produce a cell-laden hydrogel and cultured in Dulbecco’s modified Eagle’s media supplemented with 10% fetal bovine serum and 1% penicillin/streptomycin at 37 °C and 5% CO_2_ for 1, 3, and 5 days. Cell viability was determined using the live/dead cytotoxicity kit (Dojindo, Japan). Encapsulated cells were imaged under a fluorescence microscope (X71; Olympus). The proliferation of L929 cells was assessed by the Counting Kit-8 (CCK-8) method. Briefly, L929 cells were seeded into 96-well plates with a density of 1000 cells/100 μL/well and incubated for 24 h at 37 °C in a 5% CO_2_ humidified incubator to obtain a monolayer of cells. Cell medium was replaced with hydrogel extracts and further incubated for 1, 3, and 5 days. The sample solution was removed and CCK-8 reagent was added to each well and incubated for 2 h at 37 °C. The absorbance was measured using a microplate reader (SpectraMax 190, USA) at 450 nm. For each sample, five independent cultures were prepared and proliferation assays were repeated three times for each culture.

### Cell attachment test

C3H cells (C3H10T1/2, Cell bank of the Chinese Academy of Science) (5 × 10^4^ cells/hydrogel) were seeded onto the GelMA/HA-NB/LAP hydrogel coated onto coverslips. Cells were stained with phalloidin (Cytoskeleton, Inc.) and DAPI (4′,6-diamidino- 2-phenylindole, Beyotime Institute of Biotechnology, Inc., Jiangsu, China) 1, 3, and 5 days following seeding. Before staining, cells were fixed in 4% (v/v) paraformaldehyde for 20 min, permeabilized in 0.1% (w/v) Triton X-100 (Sigma-Aldrich) for 5 min, and then blocked with 1% bovine serum albumin (Sigma-Aldrich) for 30 min. Actin filaments were stained in 200 × 10^−9^ M phalloidin for 45 min and nuclei were stained in 14.3 × 10^−6^ M DAPI for 5 min. Stained cells were then imaged under a confocal microscope with a ×40 water objective (BX-FV1000, Olympus).

### In vivo degradation of hydrogels

Male rats (~ 250 g) were used in the in vivo degradation studies. All animals were treated according to the standard guidelines approved by the Zhejiang University Ethics Committee (ZJU20170969). A 1 cm incision in the mediodorsal skin of was made and a lateral subcutaneous pocket prepared. Hydrogel samples (*n* = 20; 10 × 3 mm cylinders) were implanted under sterile conditions. At designated time intervals (days 7, 14, 28, and 56), the rats were sacrificed and the samples were processed for histological analyses and biodegradation studies.

### In vivo biocompatibility

Male rats (~ 250 g) were used for the in vivo biocompatibility studies. All animals were treated according to the standard guidelines approved by the Zhejiang University Ethics Committee (ZJU20170969). A 1 cm incision was made in the rat mesodorsal epidermis and a small lateral subcutaneous pocket was prepared. Matrix hydrogel (*n* = 8), CA (*n* = 8), and Fibrin glue (*n* = 8) were implanted into the dorsal subcutaneous pockets under sterile conditions. At designated time intervals (days 7 and 14), the rats were killed and the samples were processed for histological analyses. The degree of inflammation was assessed by three expert histopathologists, under blinded experimental conditions.

### In vitro hemostasis experiments on pig livers

In vitro experiments were carried out on fresh pig livers purchased from the market to study hemostatic performance under wet and dynamic conditions. First, a 10 mm incision was pierced in pig liver and a perfusion tube was inserted with a 20 mL/min blood flow volume to mimic the heavy bleeding. The hydrogel was applied to the bleeding site and crosslinked by 365 nm UV light. The Fibrin Glue was used as control group.

### In vivo hemostasis on rabbit’s livers and arteries

To study the hemostatic properties of hydrogels in vivo, the liver lobes and femoral artery of male New Zealand white rabbits (2.5–3.0 kg, *n* = 22), and the heart and carotid artery of male BA-MA Mini-pigs (20–25 kg, *n* = 5) were used as models. All animals were treated according to guidelines approved by the Zhejiang University Ethics Committee (ZJU20170969). For liver hemostasis, a large (3 cm) incision was made in the liver using surgical scissors. The hydrogel was injected onto the incision, followed by illumination with 365 nm UV light for 3~ 5 s. During the surgery, the blood was carefully collected with filter papers at time point of 10 min. The total amount of the blood loss was determined by weighing the papers and recorded^10^. For femoral artery hemostasis, the femoral artery of rabbit was peeled from the surrounding tissues and an incision (2 mm) created by scalpel. Hemostatic forceps were then used to clamp the blood vessels. Then the hydrogel was applied to the incision site and illuminated by UV light for 3~ 5 s. After 30 s for gelation, hemostatic forceps clamped in the proximal vascular was removed to observe whether the bleeding was stopped or not. Then, the distal part of the artery was clipped to observe whether the vessel was free or not.

### Hemostatic experiments on pig carotid arteries and hearts

For hemostasis of penetrated cardiac injuries, after general anesthesia, a 6 mm inner diameter needle was used to pierce the ventriculus sinister of pig hearts (*n* = 7). For the experimental group, the defects and surrounding tissue were rapidly covered with hydrogel and irradiated by UV (*n* = 4). For the control group, the wound and surrounding tissue were first covered with Fibrin Glue, then with Surgiflo^TM^, and finally with the hydrogel, followed by UV fixation (*n* = 3). The electrocardiograph was recorded using noninvasive telemetry for large animals (Emka Technologies, France).

The carotid artery hemostasis of pigs was carried out in a similar way to the femoral artery hemostasis of rabbit, the difference being that the incision (4–5 mm) was created by needle puncture followed by full scalpel incision. Blood flow volume was tested before and after surgery using the MFV-3200 electromagnetic flow meter (Nihon Kohden, Japan). The comparative experiments with Fibrin Glue and Surgiflo^TM^ were made using carotid artery hemostasis. After these operations, one pig was sacrificed for cardiac wall section histopathology and SEM analysis, and other pigs were allowed to recover.

All animals were treated according to guidelines approved by the Zhejiang University Ethics Committee (ZJU20170969).

### Myocardial enzyme test

The levels of AST, CK, and LDH in the blood were analyzed by automatic biochemical analyzer (HITACHI 7020, Tokyo, Japan). The levels of BNP, cTn-T, and CK-MB were measured by ELISA kit (Nanjing Jiancheng Bioengineering Institute).

### Histological evaluation

After 2 weeks recovery, the pigs were sacrificed. The heart and carotid artery were surgically removed and the samples were processed for histological analyses.

### Statistical analysis

All data are presented as the mean ± SD. Differences between the values were evaluated using one-way analysis of variance (ANOVA; Tukey’s post-hoc test), except in vivo biocompatibility experiment (two-way ANOVA; Tukey’s post-hoc test). Data are presented as means with 95% confidence interval. *p* < 0.05 was considered statistically significant.

### Reporting summary

Further information on research design is available in the Nature Research Reporting Summary linked to this article.

## Supplementary information

Supplementary Information

Supplementary Movie 1

Supplementary Movie 2

Supplementary Movie 3

Supplementary Movie 4

Supplementary Movie 5

Supplementary Movie 6

Supplementary Movie 7

Description of Additional Supplementary Files

Reporting Summary

Peer Review File

Source Data

## Data Availability

The authors declare that all data supporting the findings of this study are available within the paper, its Supplementary Information, the Source Data file, or from the authors upon reasonable request.

## References

[1] Pfeifer R, Tarkin IS, Rocos B, Pape HC (2009). Patterns of mortality and causes of death in polytrauma patients—Has anything changed?. Injury.

[2] Reddy D, Muckart DJJ (2014). Holes in the heart: an atlas of intracardiac injuries following penetrating trauma. Interact. Cardiovasc. Thorac. Surg..

[3] Laurenti JB (2017). Enhanced pro-coagulant hemostatic agents based on nanometric zeolites. Micro. Mesopor Mat..

[4] Quan K (2016). Diaminopropionic acid reinforced graphene sponge and its use for hemostasis. ACS Appl. Mater. Inter..

[5] Chen Y (2016). Preparation of porous carboxymethyl chitosan grafted poly (acrylic acid) superabsorbent by solvent precipitation and its application as a hemostatic wound dressing. Mater. Sci. Eng. C..

[6] Schonauer C, Tessitore E, Barbagallo G, Albanese V, Moraci A (2004). The use of local agents: bone wax, gelatin, collagen, oxidized cellulose. Eur. Spine J..

[7] Shin M (2017). Complete prevention of blood loss with self-sealing haemostatic needles. Nat. Mater..

[8] Shin M (2015). DNA/tannic acid hybrid gel exhibiting biodegradability, extensibility, tissue adhesiveness, and hemostatic ability. Adv. Funct. Mater..

[9] Ellis-Behnke RG (2006). Nano hemostat solution: immediate hemostasis at the nanoscale. Nanomed. Nanotechnol. Biol. Med..

[10] Li J (2017). Tough adhesives for diverse wet surfaces. Science.

[11] Shin J (2015). Tissue adhesive catechol-modified hyaluronic acid hydrogel for effective, minimally invasive cell therapy. Adv. Funct. Mater..

[12] Brennan MJ, Kilbride BF, Wilker JJ, Liu JC (2017). A bioinspired elastin-based protein for a cytocompatible underwater adhesive. Biomaterials.

[13] Chang EI (2011). Vascular anastomosis using controlled phase transitions in poloxamer gels. Nat. Med.

[14] Lang N (2014). A blood-resistant surgical glue for minimally invasive repair of vessels and heart defects. Sci. Transl. Med..

[15] Annabi N (2017). Engineering a highly elastic human protein–based sealant for surgical applications. Sci. Transl. Med..

[16] Sun W (2016). Polymer-supramolecular polymer double-network hydrogel. Adv. Funct. Mater..

[17] Yang Y (2016). Tissue‐integratable and biocompatible photogelation by the imine crosslinking reaction. Adv. Mater..

[18] Campbell, P. K., Bennett, S. L., Driscoll, A., & Sawhney, A. S. *Evaluation of absorbable surgical sealants: in-vitro testing*. (Confluent Surgical, Inc., Waltham, MA, 2005).

[19] Hrabchak C (2010). Assessment of biocompatibility and initial evaluation of genipin cross-linked elastin-like polypeptides in the treatment of an osteochondral knee defect in rabbits. Acta Biomater..

[20] Annabi N (2014). 25th Anniversary article: rational design and applications of hydrogels in regenerative medicine. Adv. Mater..

[21] Park HJ (2006). Human umbilical vein endothelial cells and human dermal microvascular endothelial cells offer new insights into the relationship between lipid metabolism and angiogenesis. Stem Cell Rev..

[22] Gaharwar AK (2014). Shear-thinning nanocomposite hydrogels for the treatment of hemorrhage. ACS Nano.

[23] Sun JY (2012). Highly stretchable and tough hydrogels. Nature.

[24] Behrens AM (2014). Blood-aggregating hydrogel particles for use as a hemostatic agent. Acta Biomater..

[25] Burnett LR (2013). Hemostatic properties and the role of cell receptor recognition in human hair keratin protein hydrogels. Biomaterials.

[26] Fan Z (2014). A novel wound dressing based on Ag/graphene polymer hydrogel: effectively kill bacteria and accelerate wound healing. Adv. Funct. Mater..

[27] Lee G, Lee CK, Bynevelt M (2010). DuraSeal-hematoma concealed hematoma causing spinal cord compression. Spine.

[28] Cho AB (2009). Fibrin glue application in microvascular anastomosis: comparative study of two free flaps series. Microsurg.

[29] Apostolakis EE, Leivaditis VN, Anagnostopoulos C (2009). Sutureless technique to support anastomosis during thoracic aorta replacement. J. Cardiothorac. Surg..

[30] Liu LQ, Liu JS, Zhu MJ, Hu SX (2006). Experimental study of one-shot vascular anastomostic device for proximal vein graft anastomoses. Ann. Thorac. Surg..

[31] Nichol JW (2010). Cell-laden microengineered gelatin methacrylate hydrogels. Biomaterials.

[32] Xiao W (2011). Synthesis and characterization of photocrosslinkable gelatin and silk fibroin interpenetrating polymer network hydrogels. Acta Biomater..

[33] Fairbanks BD, Schwartz MP, Bowman CN, Anseth KS (2009). Photoinitiated polymerization of PEG-diacrylate with lithium phenyl-2,4,6-trimethylbenzoylphosphinate: polymerization rate and cytocompatibility. Biomaterials.

[34] Zhao X (2016). Photocrosslinkable gelatin hydrogel for epidermal tissue engineering. Adv. Health. Mater..

[35] Azuma K (2015). Biological adhesive based on carboxymethyl chitin derivatives and chitin nanofibers. Biomaterials.

