# Supplementary material for: A strongly adhesive hemostatic hydrogel for the repair of arterial and heart bleeds
Source: Nat Commun. 2019 May 14;10:2060. doi: 10.1038/s41467-019-10004-7 (PMC6517429; doi:10.1038/s41467-019-10004-7)
Supplement: Supplementary file 1 — Supplementary Information [file 41467_2019_10004_MOESM1_ESM.pdf]

Supplementary Information

for "A strongly adhesive haemostatic hydrogel for the repair of arterial and heart bleeds"

Hong et al.

Component	Final polymer concentration (%, w/v)	Gel point (s)	Final torsion modulus G' (Pa)	Wound closure test (kPa)	Burst pressure (mmHg)
HA-NB (1.04% substitution)/GelMA	1.25% / 5% (52.6% substitution)	≈1.384	1077 ± 55.194	22.78 ± 7.980	83.5 ± 25.171
	1.25% / 5% (85.5% substitution)		724.067 ± 24.715	13.11 ± 0.478	77 ± 12.377
HA-NB (3.27% substitution)/GelMA	1.25% / 5% (52.6% substitution)		3183.333 ± 102.051	38.19 ± 2.241	154.75 ± 26.58
	1.25% / 5% (85.5% substitution)		1842.333 ± 123.087	17.143 ± 6.337	57.5 ± 10.238
HA-NB (6.75% substitution)/GelMA	1.25% / 5% (52.6% substitution)		6146 ± 141.534	41.68 ± 6.878	190.25 ± 13.255
	1.25% / 5% (85.5% substitution)		5520.333 ± 272.107	24.757 ± 8.173	153.25 ± 23.98
HA-NB (3.27% substitution)/GelMA (52.6% substitution)	0.625% / 2.5%		692.267 ± 61.051	4.56 ± 1.006	20.75 ± 8.227
	0.625% / 5%		1834.667 ± 48.686	10.807 ± 0.480	56.25 ± 5.612
	0.625% / 7.5%		2439.667 ± 236.189	14.547 ± 2.886	80.625 ± 15.928
	1.25% / 2.5%		1372.667 ± 65.363	21.7 ± 2.953	92 ± 14.078
	1.25% / 5%		3183.333 ± 102.051	38.19 ± 2.241	154.75 ± 26.58
	1.25% / 7.5%		3342.667 ± 68.530	47.667 ± 3.723	201.5 ± 59.607
	2.5% / 2.5%		3236.333 ± 214.836	21.98 ± 1.526	131 ± 3.031
	2.5% / 5.0%		5138 ± 168.199	42.238 ± 2.735	183.938 ± 21.101
	2.5% / 7.5%		7807.333 ± 577.703	65.813 ± 3.996	214.125 ± 6.524
	2.5% / 10.0%		14516.667 ± 333.067	82.137 ± 4.005	290 ± 18.02
Cyanoacrylate (CA)	/	/	/	0	24.188 ± 4.170
Fibrin glue	/	/	/	7.063 ± 2.940	63.188 ± 23.577
Surgiflo	/	/	/	0	7.313 ± 2.831

Supplementary Table 1.

The influence of concentration and substitution ratios on the properties of the hydrogels. A table of results of a systematic study of mechanical properties of the hydrogel matrices and their resistance to rupture is shown. Strength of adhesion in the wound closure test and burst pressure increased with the substitution levels in the HA-NB matrix. While the values of final torsion modulus, adhesion strength and burst pressure all increase with rising concentrations of GelMA and HA-NB, the different percentages of each component did not markedly affect gel polymerization and the gelling point of all groups was about 1.38s.

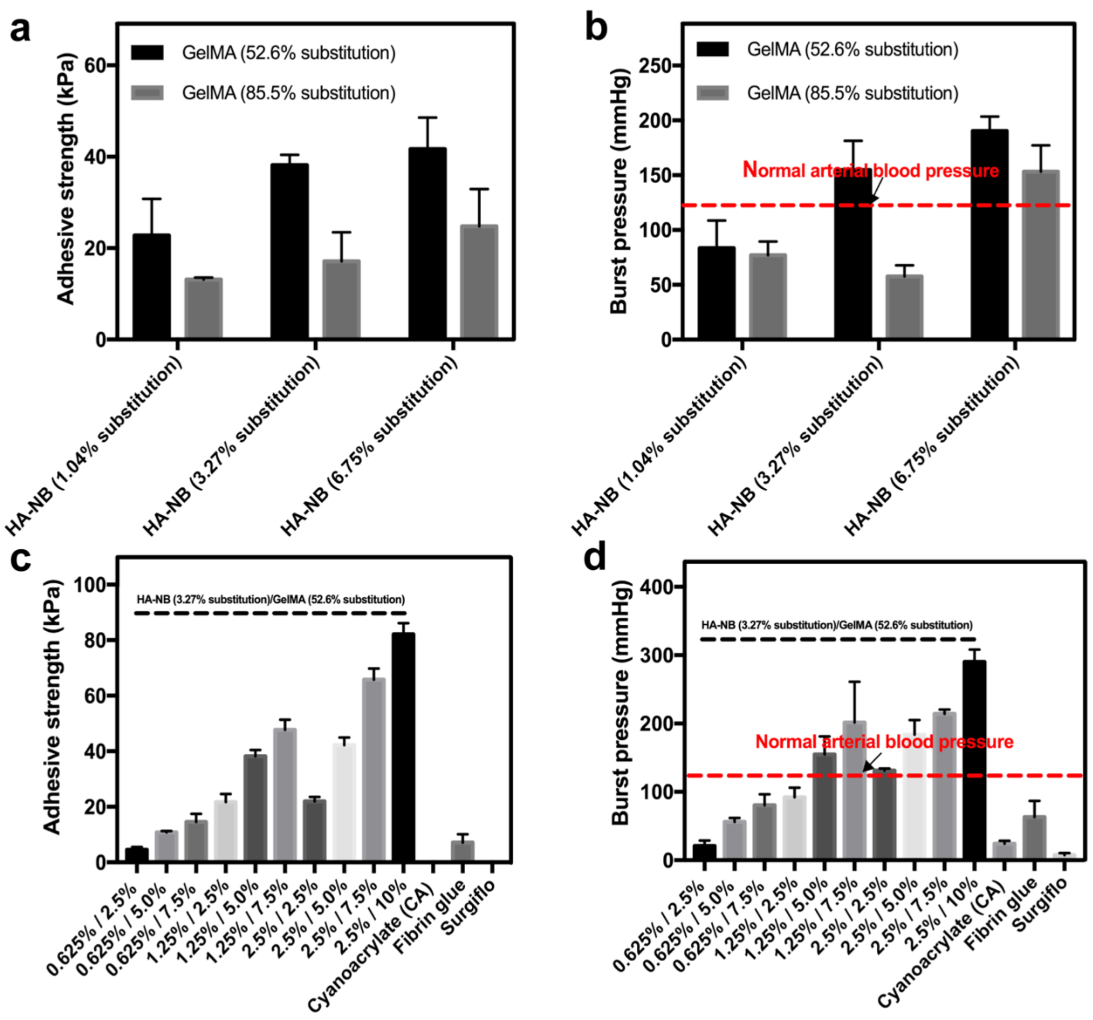

Supplementary Figure 1.

Comparison of different hydrogels with the commercially available surgical sealants. (a). Adhesion strength comparisons between different GelMA composition ratios and HA-NB substitution levels. **(b).** Burst pressure comparisons between hydrogels, with indication of normal arterial blood pressure. **(c).** Adhesive strengths of various concentrations and substitution levels in the hydrogel mixes, compared with Fibrin Glue, Cyanoacrylate glue and Surgiflo^{TM}, sealants. Cyanoacrylate and Surgiflo$^{\mathbf{TM}}$ could not be used in the adhesive strength assays because these materials do not adhere to wet surfaces. **(d).** Burst pressure comparisons between the various concentration ratio/substitution level hydrogel mixes, compared with Cyanoacrylate and Fibrin Glues and Surgiflo^{TM}. These assays essentially measure ‘hole plugging’ capacity and the three commercially available sealants can be utilized in such assays, although true adhesion is only achieved with dry surfaces. (*n* = 3 per group). Error bars, mean ± s.d.

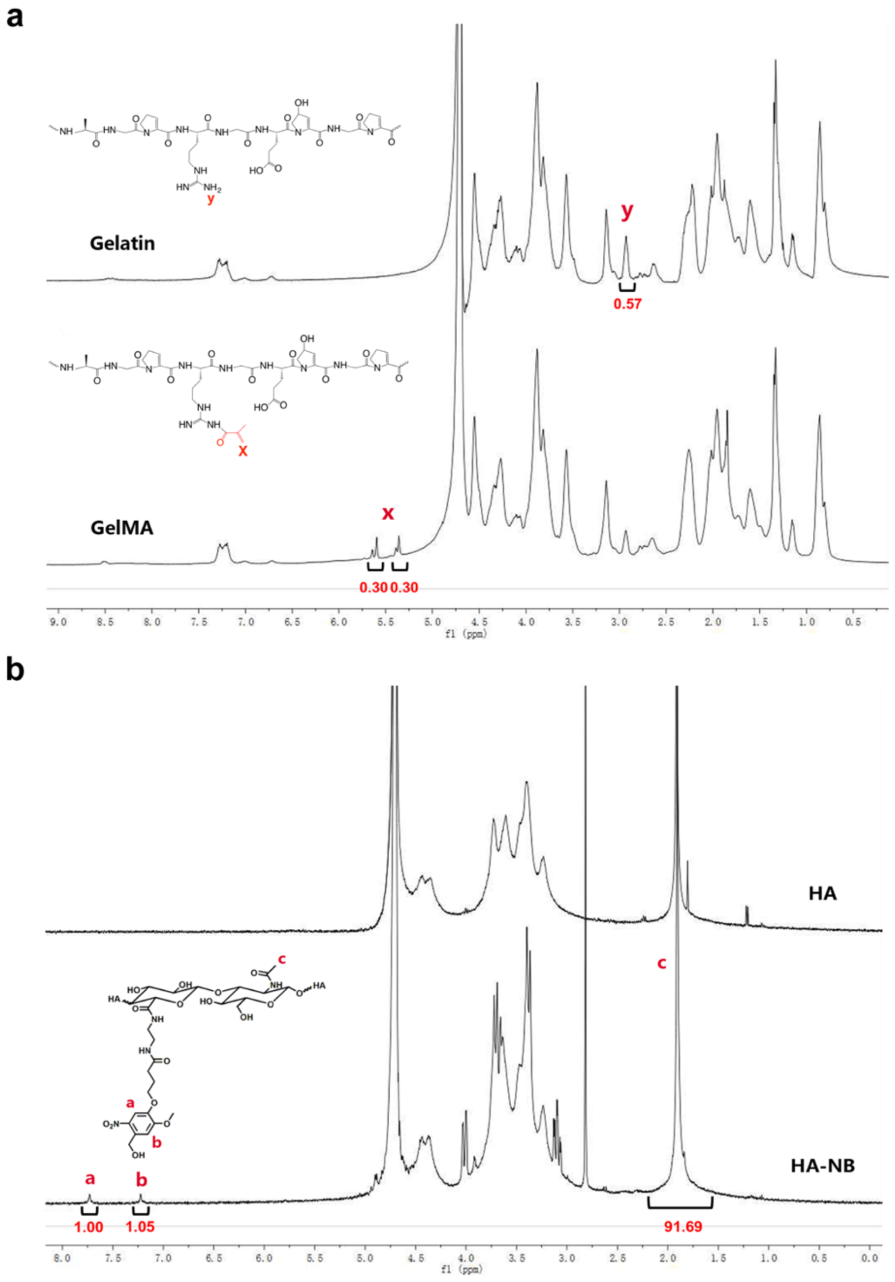

Supplementary Figure 2.

Nuclear Magnetic Resonance spectroscopic characterization of GelMA and HA-NB. (**a**) ^{1}H NMR spectra of gelatin and GelMA prepared in D_2O at room temperature. (**b**) ^{1}H NMR spectra of HA and HA-NB prepared in D_2O at room temperature. The degree of methacrylation of gelatin and the degree of NB substitution of hyaluronic acid were calculated from integrating the peak areas in the H^1 NMR spectra as follows;

Degree of methacrylation (DM) = $\frac{I_x}{I_y} \times 100$.   (1)

Degree of NB (DN) = $\frac{3I_a}{I_c} \times 100\%$.   (2)

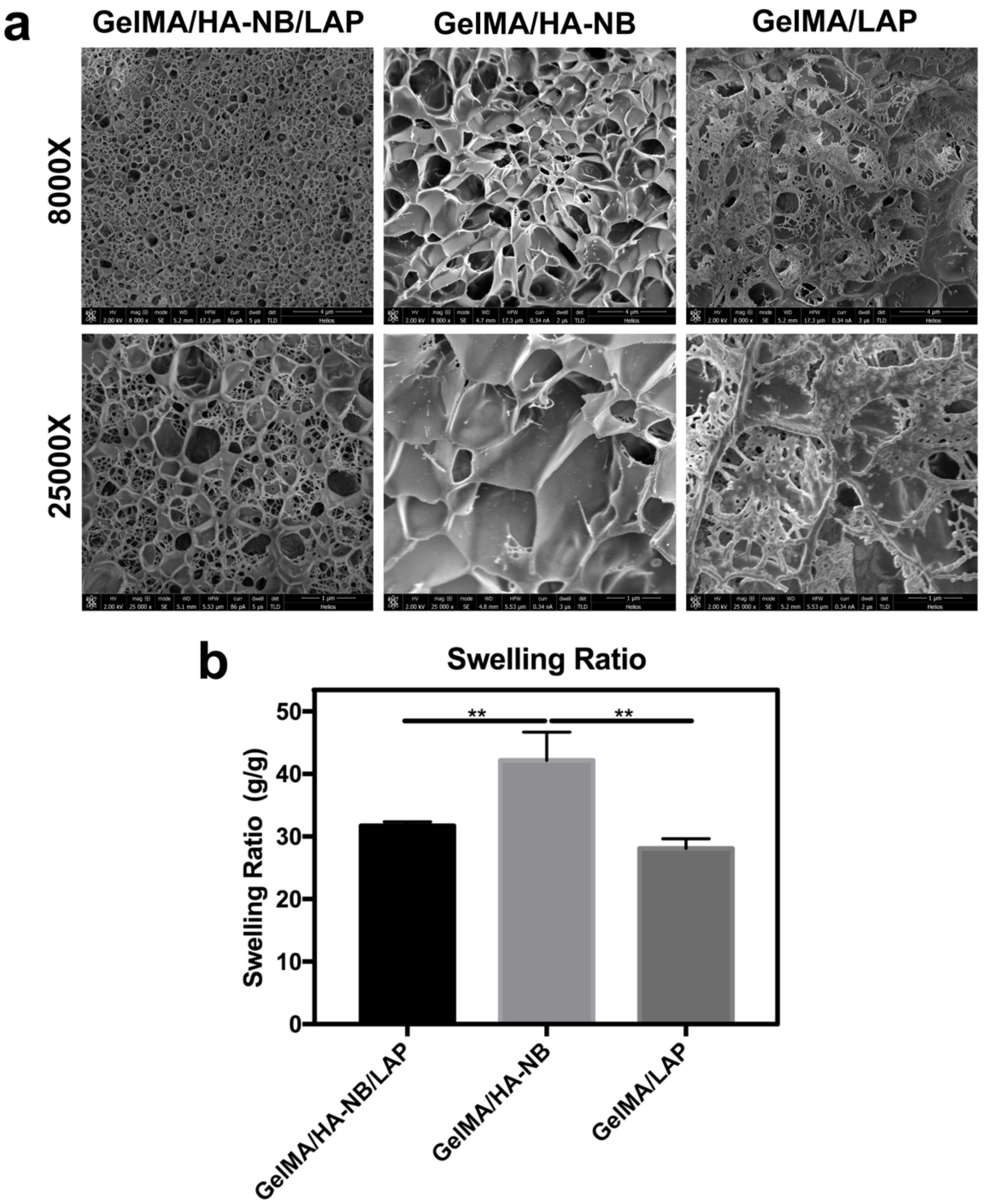

Supplementary Figure 3.

Microstructure and swelling ratios characterization of different hydrogels. (a) cryo-SEM images of GelMA/HA-NB/LAP, GelMA/HA-NB and GelMA//LAP hydrogels. Scale bar:4 μm (8000X); 1 μm (25000X). **(b**) Swelling ratios of different hydrogels (*n* = 3 per group) after 24h incubation in PBS at 37 ℃. Error bars, mean ± s.d. (**, $p < 0.01$, ****, $p < 0.0001$, One-Way analysis of variance, ANOVA, Tukey's post hoc test).

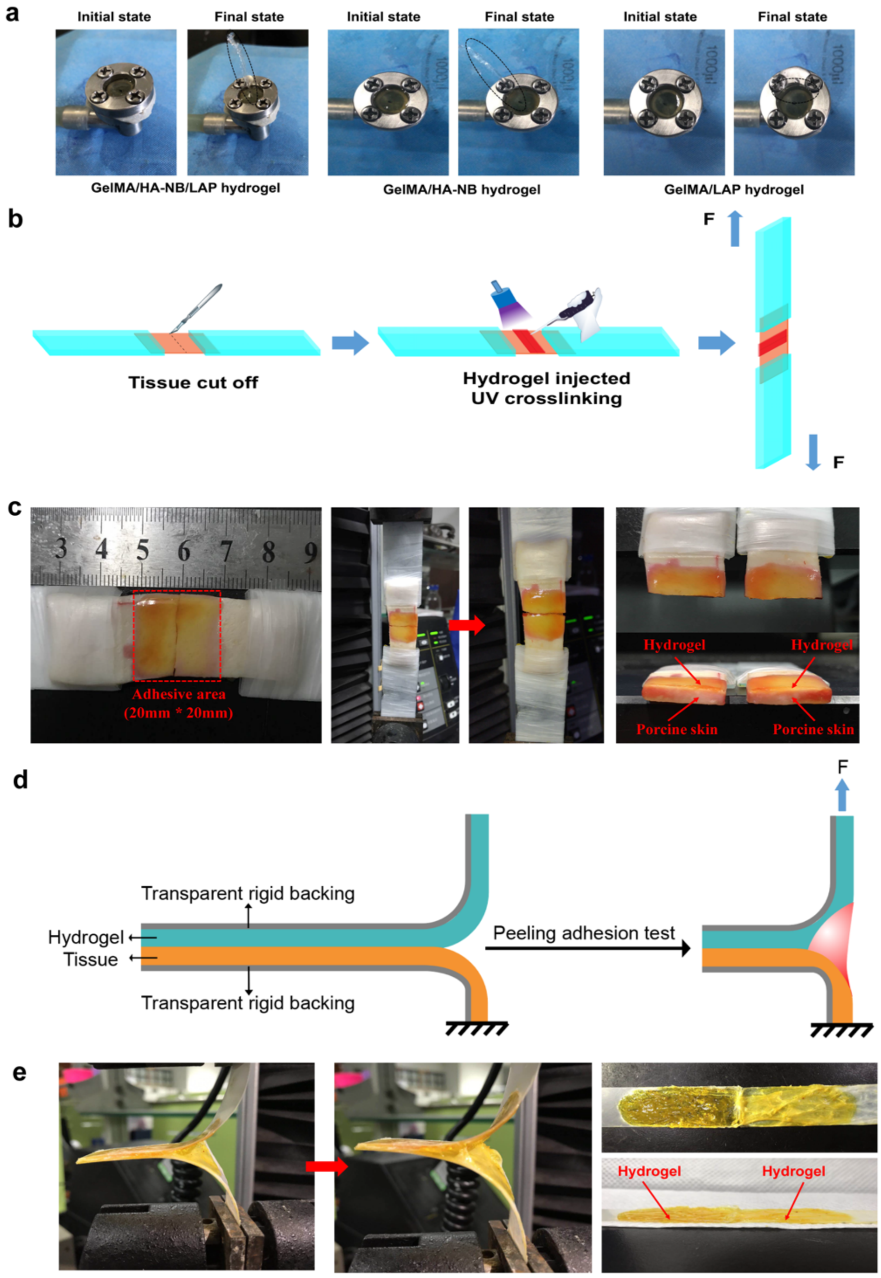

Supplementary Figure 4.

Adhesion property assays using hydrogel bonded to porcine skin. (**a**)The failure positions of GelMA/HA-NB/LAP, GelMA/HA-NB and GelMA/LAP after burst pressure testing. The assays shown in (**b-e**) were devised to test the strength of binding between hydrogel and biological tissue (porcine skin) based on the mechanical strength of resistance to peeling apart of the surfaces, and were measured using an **Instron 5543** mechanical stretch testing instrument (**b**). Schematic of the wound closure test. (**c**). The images of the adhesion stretch resistance measurement process. (**d**). Schematic of the peeling adhesion test. (**e**). Images of the point of separation (i.e. adhesion failure), taken during the peeling adhesion test. (n = 3 per group)

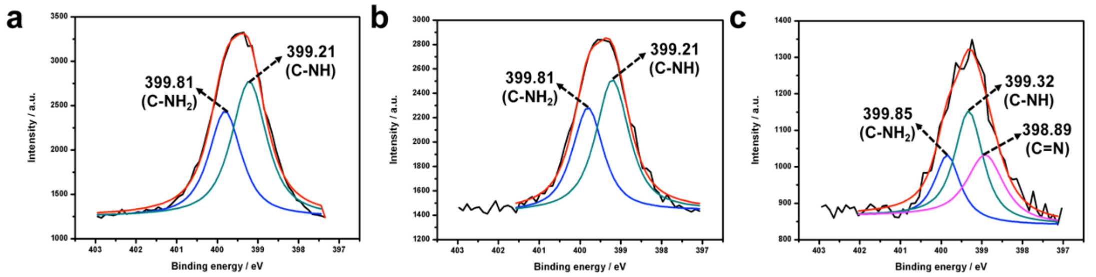

Supplementary Figure 5.

X-Ray photon spectroscopy (XPS) of HA-NB bonding to porcine sausage skin casings. XPS is limited to surface imaging, penetrating ~ 1–10 nm into the sample surface and thus cannot be used directly with thicker hydrogels. To investigate chemical bonding reactions between biological tissue and the hydrogel, we analysed HA-NB solution application to commercially available sausage skins. (**a**) X ray photon spectrum of sausage skin alone. (**b**). X ray photon spectrum of sausage skin surface-wetted with non-photo-illuminated HA-NB. (**c**). X ray photon spectrum of sausage skin + HA-NB after UV irradiation for 3 seconds. Spectra A and B are almost the same, the bond energies of C-NH bonds and C-NH_2 bonds being located at 399.21 eV and 399.81 eV, respectively. Peak shifts were observed after the UV irradiation treatment, the bond energies of the peptide -C-NH- and amino group C-NH_2 shifting to 399.32 and 399.85 respectively with the appearance of a C=N bond peak located at 398.89 eV revealing the UV-induced formation of Schiff bases[1,2].

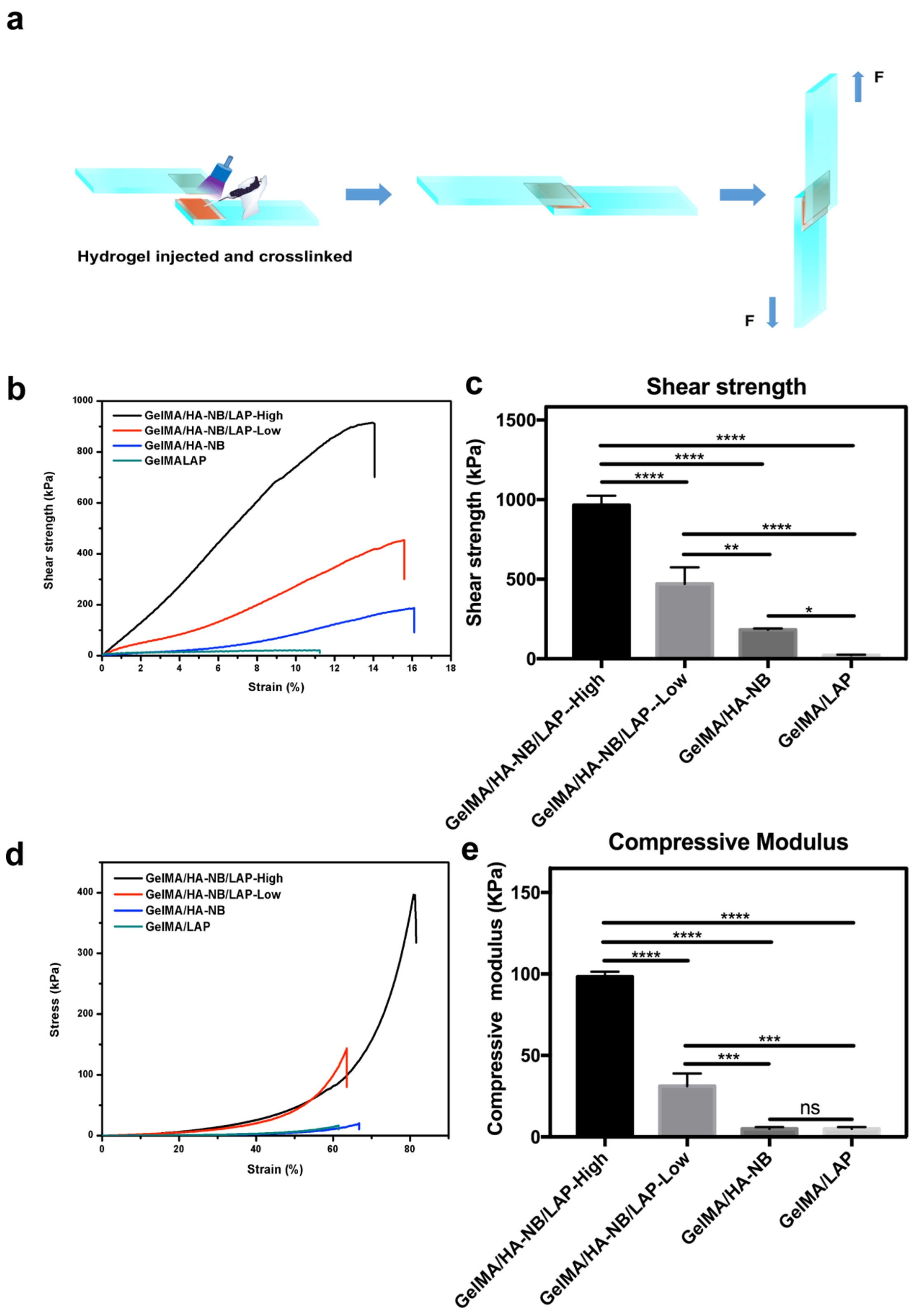

Supplementary Figure 6.

***In vitro* shear tests and mechanical compression testing of different hydrogels.** (**a**) Schematic of the modified test method used for the overlapping shear test, measured using the **Instron 5543** mechanical force testing instrument. (**b**) Representative strain-stress curves for overlapping shear tests. (**c**) The average shear resistance strength of different hydrogels. The **Instron 5543** mechanical

force testing instrument was also used to measure resistance to compressive failure of the hydrogels (*n* = 4 per group). (**d**) The elastic compressibility of different hydrogels, measured until crush failure. (**e**) Compressive modulus (15-25% strain pressure) of different hydrogels (*n* = 3 per group). Error bars, mean ± s.d. (ns: not significant, ***, $p < 0.001$, ****, $p < 0.0001$, One-Way analysis of variance, ANOVA, Tukey's post hoc test).

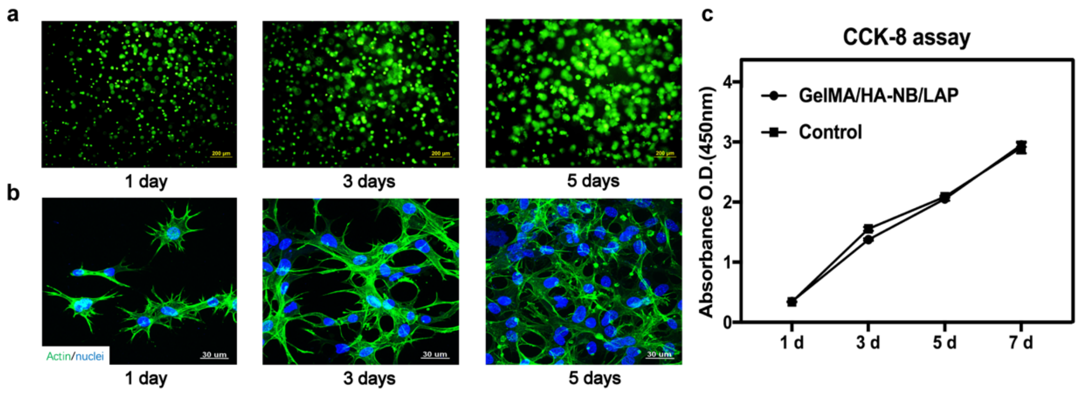

Supplementary Figure 7.

Evaluation of *in vitro* biocompatibility of GelMA/HA-NB/LAP hydrogels with live cell cultures.

(**a**) Live-Dead staining of L929 fibroblasts encapsulated in the hydrogels after 1, 3 and 5 days of incubation. L929 fibroblasts were stained with calcein AM to detect living cells (green) and ethidium homodimer-1 to detect dead cells (red). Scale bar: 200 µm.

(**b**) F-actin and DAPI nuclear staining of C3H cells seeded on the surface of the hydrogel after 1, 3, and 5 days of incubation. Note cell spreading, proliferation and growth into the hydrogel matrix. Scale bar: 30 µm.

(**c**) The cytotoxicity of this hydrogel to L929 fibroblasts after incubation for 1, 3, 5 and 7 days. (n = 3 per group). Error bars, mean ± s.d.

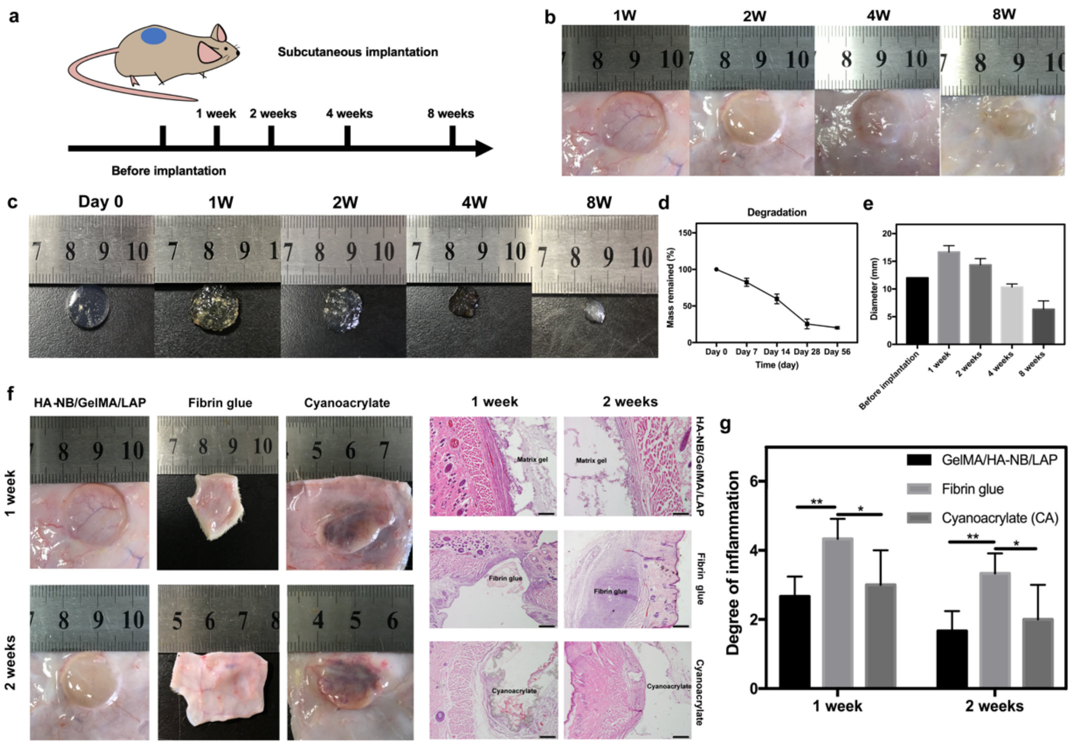

Supplementary Figure 8.

Biocompatibility testing by subcutaneous implantation.

(**a**). Schematic of subcutaneous implantation regime, using the 5% GelMA, 1.25% HA-NB (Low concentration gel). (**b**). Macroscopic appearance of excised hydrogel implants after 7, 14, 28, and 56 days of implantation. (**c**). Hydrogel appearance, shown before implantation and then at 7, 14, 28, and 56 days post-implantation. (**d**). The *in vivo* degradation profile of matrix hydrogel gel over time, based on dry weight measurements. **(e)**. The diameter of hydrogels implants before the procedure and then after 7, 14, 28, and 56 days of implantation. (n = 5 per group). **(f).** Macroscopic view of explanted GelMA/HA-NB/LAP, Fibrin Glue and Cyanoacrylate Glue implants, after 7 and 14 days of implantation and hematoxylin/eosin staining of tissue sections containing the different implants, after 7 and 14 days of implantation. Scale bar: 500 µm. (**g**) Degree of inflammation, determined by three experienced pathologists, for the three groups: GelMA/HA-NB/LAP, Fibrin Glue and Cyanoacrylate Glue at 7 and 14 days post-implantation (0=normal, 1=very mild, 2=mild, 3=moderate, 4=severe, 5=very severe). Error bars, mean ± s.d. (*, $p < 0.05$, **, $p < 0.01$, Two-Way analysis of variance, ANOVA, Tukey's post hoc test). (n = 4 per group). These slides were read blinded, the operator being unaware which treatment had been used.

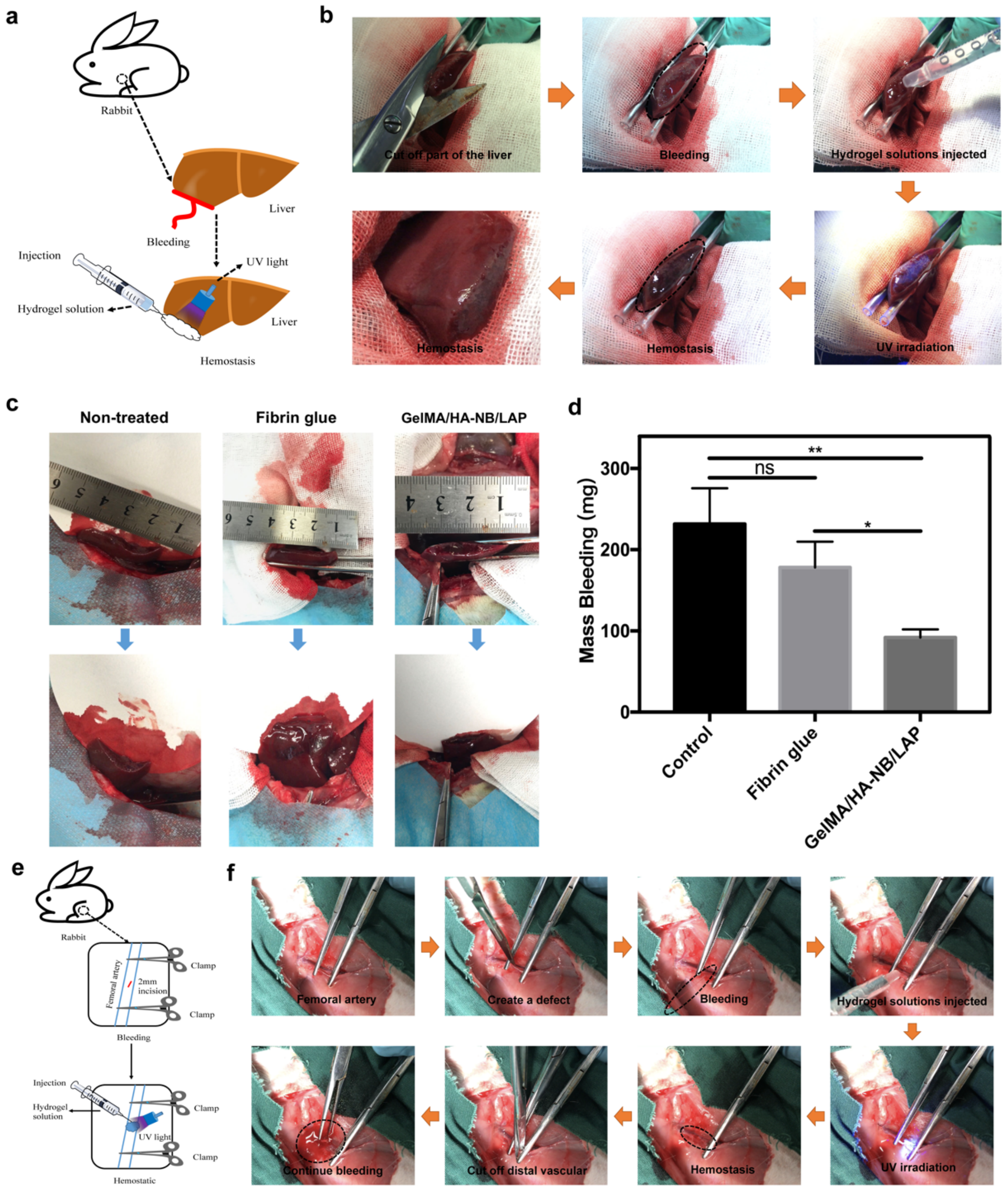

Supplementary Figure 9.

Rapid *in vivo* hemostasis following rabbit liver resection and femoral artery lesions.

(a & b). A section of liver was exposed from the abdominal cavity of a rabbit under anesthesia and gripped by tweezers. **(c).** A part of the liver lobe was excised (3 cm), leading to wound bleeding. The wound was then covered by the precursor of the matrix gel and UV irradiated for 3-5 seconds. After the hydrogel was applied and fixed, bleeding ceased. **(d).** Comparison of the amounts of blood lost with control, fibrin glue and hydrogel treatment. This *in vivo* hemostasis experiment has been successfully repeated in rabbits >10 times, demonstrating the reliability of the hydrogel treatment. The amount of blood loss in the hydrogel treatments was consistently and significantly less than in control and Fibrin Glue treated groups. Error bars, mean ± s.d. (ns: no significance, *, $p < 0.05$, **, $p < 0.01$, One-Way analysis of variance, ANOVA, Tukey's post hoc test). (n=12). **(e & f).** The femoral artery of the anaesthetized rabbit was first separated from the surrounding tissues and two hemostatic forceps were used to clamp the artery prior to making an incision. When a 1~2 mm

arterial incision was made, blood pumped out when the forceps at the cardiac proximal end was removed. After reapplying the forceps, the hydrogel was applied to the incision site and irradiated with UV light for 3~5 s. 30 s after fixation the forceps were removed and no bleeding was observed. Then, the distal end was clipped using surgical scissors and heavy arterial bleeding recommenced, indicating the intactness and viability of the femoral artery after the gel treatment. This surgical procedure has been repeated 10 times (*n*=10), with similar results, demonstrating the reliability of the hydrogel repair methodology.

Supplementary References:

1. Hu et al, Probing the chemical structure of monolayer covalent-organic frameworks grown via Schiff-base condensation reactions. *Chem. Commun*. **52**, 9941-9944 (2016).
2. Zhuang et al. Graphene Coupled Schiff‐base Porous Polymers: Towards Nitrogen‐enriched Porous Carbon Nanosheets with Ultra-high Electrochemical Capacity. *Advanced Materials* **26**, 3081-3086 (2014).
