# Supplementary material for: A strongly adhesive hemostatic hydrogel for the repair of arterial and heart bleeds
Source: Nat Commun. 2019 May 14;10:2060. doi: 10.1038/s41467-019-10004-7 (PMC6517429; doi:10.1038/s41467-019-10004-7)
Supplement: Supplementary file 9 — Description of Additional Supplementary Files [file 41467_2019_10004_MOESM9_ESM.docx]

**Supplementary Movie 1.**

***Ex vivo* simulated hemostasis test.** GelMA/HA-NB/LAP hydrogel was used to seal and stop bleeding of an incision in an exised pig liver with ~20 ml/min blood flow volume. (*n*=3)

**Supplementary Movie 2**

***Ex vivo* simulated hemostasis test.** Fibrin glue was used as a control group to seal and stop bleeding of an incision in exised pig liver with ~20 ml/min blood flow volume. (*n*=3)

**Supplementary Movie 3**

***In vivo* hemostasis test using a pig carotid artery defect model.** GelMA/HA-NB/LAP hydrogel was used for rapid hemostasis of pig carotid artery with a 4~5 mm bleeding incision. (*n*=3)

**Supplementary Movie 4**

***In vivo* hemostasis test using a pig carotid artery defect model to compare Fibrin Glue and Surgiflo^TM^ performance.** Fibrin Glue and Surgiflo^TM^ were used as to compare their hemostasis of pig carotid artery with a 4~5 mm incision. (*n*=3)

**Supplementary Movie 5**

***In vivo* hemostasis test using a pig penetrated cardiac injury model.** GelMA/HA-NB/LAP hydrogel was used for rapid hemostasis of pig heart with a 6-mm whole-layer cardiac penetration hole. (*n*=3)

**Supplementary Movie 6**

***In vivo* hemostasis test using a pig penetrated cardiac injury model of extremely serious bleeding.** GelMA/HA-NB/LAP hydrogel was used for rapid hemostasis of pig heart with a 6-mm whole-layer cardiac penetration hole leading to extremely serious bleeding. (*n*=3)

**Supplementary Movie 7**

***In vivo* hemostasis test in a pig penetrated cardiac injury model using Fibrin Glue and Surgiflo^TM^.** Fibrin Glue and Surgiflo^TM^ were used as comparisons for hemostasis of pig heart with a 6-mm whole-layer cardiac penetration hole. (*n*=3)
