# Supplementary material for: A strongly adhesive hemostatic hydrogel for the repair of arterial and heart bleeds
Source: Nat Commun. 2019 May 14;10:2060. doi: 10.1038/s41467-019-10004-7 (PMC6517429; doi:10.1038/s41467-019-10004-7)
Supplement: Supplementary file 10 — Reporting Summary [file 41467_2019_10004_MOESM10_ESM.pdf]

nature research

Corresponding author(s): Linyong Zhu, Shufang Zhang and Hongwei Ouyang

Last updated by author(s): Mar 22, 2019

Reporting Summary

Nature Research wishes to improve the reproducibility of the work that we publish. This form provides structure for consistency and transparency in reporting. For further information on Nature Research policies, see Authors & Referees and the Editorial Policy Checklist.

Statistics

For all statistical analyses, confirm that the following items are present in the figure legend, table legend, main text, or Methods section.

n/a	Confirmed	
☐	☒	The exact sample size (*n*) for each experimental group/condition, given as a discrete number and unit of measurement
☐	☒	A statement on whether measurements were taken from distinct samples or whether the same sample was measured repeatedly
☐	☒	The statistical test(s) used AND whether they are one- or two-sided<br>*Only common tests should be described solely by name; describe more complex techniques in the Methods section.*
☐	☒	A description of all covariates tested
☐	☒	A description of any assumptions or corrections, such as tests of normality and adjustment for multiple comparisons
☐	☒	A full description of the statistical parameters including central tendency (e.g. means) or other basic estimates (e.g. regression coefficient) AND variation (e.g. standard deviation) or associated estimates of uncertainty (e.g. confidence intervals)
☐	☒	For null hypothesis testing, the test statistic (e.g. *F*, *t*, *r*) with confidence intervals, effect sizes, degrees of freedom and *P* value noted<br>*Give P values as exact values whenever suitable.*
☒	☐	For Bayesian analysis, information on the choice of priors and Markov chain Monte Carlo settings
☐	☒	For hierarchical and complex designs, identification of the appropriate level for tests and full reporting of outcomes
☐	☒	Estimates of effect sizes (e.g. Cohen's *d*, Pearson's *r*), indicating how they were calculated

Our web collection on statistics for biologists contains articles on many of the points above.

Software and code

Policy information about availability of computer code

Data collection	Image acquisition of fluorescence experiments was performed using fluorescence microscope (X71; Olympus, Tokyo, Japan) and confocal microscope with a 60× water objective (BX-FV1000, Olympus, Tokyo, Japan), image analyses: FV31S-SW, v2.1.1.98.
Data analysis	Chemical structural formula was drawn using the Chemical Draw, v15.0.0.106.<br><br>NMR data analysis was performed using mestrenova, v9.0.1.<br><br>Blood flow volume analysis was performed using MedLab, v6.6.6.<br><br>All statistical analyses were performed using Origin 8.0 and GraphPad Prism 7.0.

For manuscripts utilizing custom algorithms or software that are central to the research but not yet described in published literature, software must be made available to editors/reviewers. We strongly encourage code deposition in a community repository (e.g. GitHub). See the Nature Research guidelines for submitting code & software for further information.

Data

Policy information about availability of data

All manuscripts must include a data availability statement. This statement should provide the following information, where applicable:

- Accession codes, unique identifiers, or web links for publicly available datasets
- A list of figures that have associated raw data
- A description of any restrictions on data availability

A full data availability statement is included in the manuscript.

Field-specific reporting

Please select the one below that is the best fit for your research. If you are not sure, read the appropriate sections before making your selection.

☒ Life sciences ☐ Behavioural & social sciences ☐ Ecological, evolutionary & environmental sciences

For a reference copy of the document with all sections, see nature.com/documents/nr-reporting-summary-flat.pdf

Life sciences study design

All studies must disclose on these points even when the disclosure is negative.

Sample size	No statistical method was used to predetermine sample size.<br>For in vitro experiments:<br>1. Scanning electron microscopy analysis, 3 samples each group were used.<br>2. Swelling ratio analysis, 3 samples (diameter=10mm, high=5mm cylinders) each group were used.<br>3. Rheological studies, 3 samples (200 µl pregel solution) each group were used.<br>4. In vitro lap shear test, 4 samples (200 µl pregel solutions were added to the gelatin-coated regions (10 mm × 15 mm)) were used for each group.<br>5. Bursting Pressure Test, 3 samples (500 µl pregel solutions were added to device (inner diameter=12 mm, high= 5 mm), and the thickness of the formed samples are about 4.4 mm) were used for each group.<br>6. In vitro hemostasis experiments, 3 samples (1 cm incisions were pierced in pig livers) were used for each group.<br>7. Wound closure tests, the skin sample dimensions were 30 mm × 10 mm, hydrogel solutions (1mL) were injected onto the desired adhesion zone (20 × 20mm) .<br>8. Compression test, samples were prepared in the molds for compression tests (10 mm in diameter and 5 mm in depth).<br>For in vivo experiments:<br>1. In Vivo Degradation, hydrogel samples (n = 20; diameter=10 mm, high=3 mm cylinders) were implanted under sterile conditions.<br>2. In vivo hemostasis experiments, for rabbit (n=22), a 2 mm incision was made in the femoral artery and 200 µl pregel solutions were added to cover the incision. For pig (n=7), 4~5 mm defects were created in the carotid artery of pigs, and a 6 mm inner diameter needle was used to pierce the ventriculus sinister of pig hearts, 0.5-2.5 ml pregel solutions were added to cover the incision (7 pigs were used, 6 pigs fed for 2 weeks after operation while 1 pig was sacrificed for scanning electron microscopy analysis).
Data exclusions	No animal was excluded from studies. For the pig experiment, 7 pigs were used, 6 pigs were fed for 2 weeks after operation while 1 pig was sacrificed for scanning electron microscopy analysis after operation.
Replication	All attempts at replication were successful.
Randomization	Currently, there is no gel materials or hemostatic agent can deal with these fatal bleeding situations, therefore, we are only able to evaluate the efficacy of our gel material rather than setting randomized animal experiments with control group.
Blinding	During the data collection and/or analysis, the researchers that took the blood samples, ECG data and performed the physiological index analysis were blind to groups allocation, i.e, they were not the researchers that performed the in vivo experiments.

Reporting for specific materials, systems and methods

We require information from authors about some types of materials, experimental systems and methods used in many studies. Here, indicate whether each material, system or method listed is relevant to your study. If you are not sure if a list item applies to your research, read the appropriate section before selecting a response.

Materials & experimental systems

n/a	Involved in the study	
☐	☒	Antibodies
☐	☒	Eukaryotic cell lines
☒	☐	Palaeontology
☐	☒	Animals and other organisms
☒	☐	Human research participants
☒	☐	Clinical data

Methods

n/a	Involved in the study	
☒	☐	ChIP-seq
☒	☐	Flow cytometry
☒	☐	MRI-based neuroimaging

Antibodies

Antibodies used	No individual antibody was used in our experiments except the following commercial Kits:<br>Brain natriuretic peptide Assay Kit (Nanjing Jiancheng bioengineering institute)<br>Creatine kinase MB isoenzyme Assay Kit (Nanjing Jiancheng bioengineering institute)<br>Cardiac troponin Assay Kit (Nanjing Jiancheng bioengineering institute)

Validation	Antibodies were validated by using a positive control.

Eukaryotic cell lines

Policy information about cell lines

Cell line source(s)	Mouse embryonic fibroblasts(C3H10T1/2, Cell bank of the Chinese Academy of Science)<br>Mouse fibroblast cells (L929, Cell bank of the Chinese Academy of Science)
Authentication	Functional and molecular authentication are assessed using a panel of antibody and qRT-PCR markers.
Mycoplasma contamination	All cell lines tested negative for mycoplasma contamination.
Commonly misidentified lines<br>(See ICLAC register)	No cell lines used in this study were found in the database of commonly misidentified cell lines that is maintained by ICLAC and NCBI Biosample.

Animals and other organisms

Policy information about studies involving animals; ARRIVE guidelines recommended for reporting animal research

Laboratory animals	Male New Zealand white rabbits (2.5-3.0 kg, n=22, Shanghai Jiagan biotechnology co. LTD) and male BA-MA Mini-pig (20-25 kg, n=7, Shanghai Jiagan biotechnology co. LTD) were used for all our experiments.
Wild animals	N/A
Field-collected samples	N/A
Ethics oversight	All animals were treated according to guidelines approved by the Zhejiang University Ethics Committee (ZJU20170969)

Note that full information on the approval of the study protocol must also be provided in the manuscript.
