# Supplementary material for: A strongly adhesive hemostatic hydrogel for the repair of arterial and heart bleeds
Source: Nat Commun. 2019 May 14;10:2060. doi: 10.1038/s41467-019-10004-7 (PMC6517429; doi:10.1038/s41467-019-10004-7)
Supplement: Supplementary file 11 — Peer Review File [file 41467_2019_10004_MOESM11_ESM.pdf]

Reviewers' Comments:

Reviewer #1:
Remarks to the Author:
The authors have addressed all of my feedback - the paper should be published as is.

We would like to thank the reviewers for their thoughtful and constructive comments regarding our manuscript.

Reviewer #1:
Remarks to the Author:
The authors have addressed all of my feedback - the paper should be published as is.

Response: We appreciate the reviewer's positive comments.
